# Draft genome of the most devastating insect pest of coffee worldwide: the coffee berry borer, *Hypothenemus hampei*

**DOI:** 10.1038/srep12525

**Published:** 2015-07-31

**Authors:** Fernando E. Vega, Stuart M. Brown, Hao Chen, Eric Shen, Mridul B. Nair, Javier A. Ceja-Navarro, Eoin L. Brodie, Francisco Infante, Patrick F. Dowd, Arnab Pain

**Affiliations:** 1 Sustainable Perennial Crops Laboratory, U. S. Department of Agriculture, Agricultural Research Service, Bldg. 001, Beltsville, Maryland 20705 USA; 2 NYU Center for Health Informatics and Bioinformatics, New York University School of Medicine, New York, New York 10016 USA; 3 Pathogen Genomics Laboratory, Computational Bioscience Research Center, King Abdullah University of Science and Technology, Thuwal-Jeddah, Saudi Arabia; 4 Ecology Department, Earth Sciences Division, Lawrence Berkeley National Laboratory, Berkeley, California 94720 USA; 5 El Colegio de la Frontera Sur (ECOSUR), Carretera Antiguo Aeropuerto Km. 2.5, Tapachula, 30700 Chiapas, México; 6 Crop Bioprotection Research Unit, U. S. Department of Agriculture, Agricultural Research Service, National Center for Agricultural Utilization Research, Peoria, Illinois 61604 USA

## Abstract

The coffee berry borer, *Hypothenemus hampei*, is the most economically important insect pest of coffee worldwide. We present an analysis of the draft genome of the coffee berry borer, the third genome for a Coleopteran species. The genome size is ca. 163 Mb with 19,222 predicted protein-coding genes. Analysis was focused on genes involved in primary digestion as well as gene families involved in detoxification of plant defense molecules and insecticides, such as carboxylesterases, cytochrome P450, gluthathione S-transferases, ATP-binding cassette transporters, and a gene that confers resistance to the insecticide dieldrin. A broad range of enzymes capable of degrading complex polysaccharides were identified. We also evaluated the pathogen defense system and found homologs to antimicrobial genes reported in the *Drosophila* genome. Ten cases of horizontal gene transfer were identified with evidence for expression, integration into the *H. hampei* genome, and phylogenetic evidence that the sequences are more closely related to bacterial rather than eukaryotic genes. The draft genome analysis broadly expands our knowledge on the biology of a devastating tropical insect pest and suggests new pest management strategies.

Worldwide, the genus *Hypothenemus* (Coleoptera: Curculionidae, Scolytinae) consists of 181 described species, of which only *H. hampei* (Ferrari), commonly known as the coffee berry borer, is considered an important agricultural pest^1^. The insect is present in most coffee-producing countries and damage is initiated by a 1.6–1.9 mm long adult colonizing female, which bores a hole in the coffee berry and deposits eggs in galleries within the two seeds inside the berry^1^. Upon hatching, larvae consume the seeds causing significant losses in yield and quality. Yearly losses in Brazil, the world’s top coffee producer, have been estimated at US$215–358 million^2^. The insect exhibits sibling mating inside the coffee berry, with females being preponderant, and males never leave the berry^1^. Even though the insect has been the subject of study for more than 100 years^3^, it still remains a major threat to coffee production and no consistently reliable pest management strategies are available to manage the insect.

We have sequenced the genome of female coffee berry borers in an attempt to gain a better understanding of the basic biology of the insect. Only two other Coleopteran genomes have been published to date: the red flour beetle, *Tribolium castaneum*^4^ (Tenebrionidae), and the mountain pine beetle, *Dendroctonus ponderosae*^5^, an insect in the same subfamily (Scolytinae) as the coffee berry borer. Analysis was focused on genes that could reveal important aspects of the insect’s biology, including some that might be useful in developing novel pest management strategies.

## Results and Discussion

### Gene Content and Draft Assembly

After quality filtering, a total of 62 Gbp of sequence data (36 Gbp from a 350 bp insert library and 26 Gbp from a 550 bp insert library) was used for genome assembly. With an estimated genome size of 200 Mb used for the assembly software (based on the *D. ponderosae* genome size of 204 Mb^5^), this corresponds to an average depth of coverage of 180-fold. Sequence reads were assembled using SOAPdenovo2, resulting in 163 Mb in scaffolds (see Table 1 and Table S1 for assembly statistics). The scaffolds N50 was 44.7 Kb and the contigs N50 was 10.5 Kb (Table 1). Nuñez *et al.*^6^ reported an estimated coffee berry borer genome size of 170–180 Mb with 20,653 unigenes, while Benavides *et al.*^7^ reported a 194 Mb genome, with ~20,500 unigenes. RepeatMasker analysis shows that only 2.7% of the assembled genome contains identifiable repeats and low complexity regions, which is very low for an arthropod genome, suggesting that repeated sequences may be underrepresented in the assembly.

### Transcriptome

RNA-seq reads were assembled into *de novo* transcripts using the SOAPdenovo-Trans software^8^. These predicted transcripts were functionally annotated by BLASTx similarity search against the NCBI non-redundant protein database. An additional set of “genome guided” predicted transcripts was produced from a combination of RNA-seq and genomic data using the TopHat/Cufflinks software package. RNA-seq reads were aligned to the genomic contigs using TopHat2^9^, and Cufflinks2^10^ was used for transcript assembly, yielding a set of 15,546 predicted transcripts. The Cufflinks method also produces gene expression values in units of Fragments per Kilobase per Million reads (FPKM) for each predicted transcript.

### Gene Prediction

Genes were predicted on the draft genome assembly with the PASA software system^11^ using a combination of gene expression, protein homology, and *ab inito* gene prediction. *Ab initio* gene predictions on the genomic assemblies were made with GeneMark.hmm-ET^12^. Potential protein-coding regions on the DNA assemblies were identified by tBLASTn similarity with a set of non-redundant conserved proteins from the UniProt Knowledgebase (UniRef90^13^). The transcripts built from assembly of RNA-seq reads by SOAPdenovo-Trans were aligned to the genome assembly with BLAT^14^. The GeneMark models, BLAST similarity, and RNA-seq gene expression information was combined using EVidenceModeler^11^, which produced a set of 20,301 gene models (predicted genes) and translated predicted proteins.

Predicted proteins were screened for similarity to bacterial proteins in GenBank with blastp, leading to the removal of 1,079 proteins as probable bacterial contaminants. The final set of 19,222 *H. hampei* predicted proteins are supported by GenMark HMM models, Cufflinks gene models, *de novo* assembled RNA-seq transcripts, and homology to known GenBank proteins.

One method to estimate the completeness of a genome assembly is to identify orthologs of highly conserved proteins. Using CEGMA (Core Eukaryotic Genes Mapping Approach), a method to identify highly conserved eukaryotic proteins using the NCBI KOGs database^15^, we aligned the 457 *Drosophila* CEGMA core proteins to the set of coffee borer proteins predicted from the draft genome. All 457 core proteins had significant BLAST matches, with 455 having e-values lower than 1e^−20^. This suggests that our gene collection for the coffee berry borer is nearly complete, at least for these ubiquitously expressed conserved genes.

### Non-coding RNA (ncRNA)

Functional ncRNA sequences were predicted on the *H. hampei* genome using the Infernal 1.1 software package^16^ and the Rfam database (http://rfam.xfam.org)^17^. A total of 1,085 high quality matches (e-value ≤0.01) were found, with the most abundant classes being 558 microRNAs, 181 snoRNAs, and 64 tRNAs. Sequences similar to ribozymes and CRISPR direct repeat elements were also detected. Predicted ncRNA loci are listed in Table S2.

### Biological Function of Predicted Proteins

**Figure 1:**
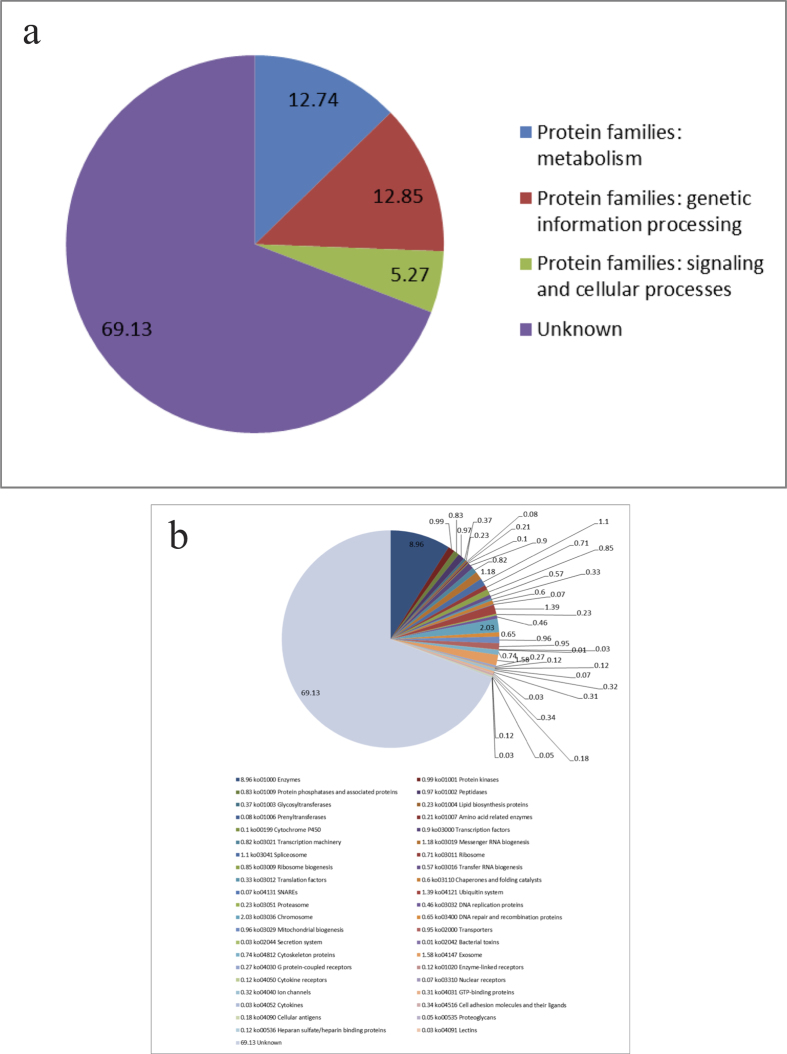
KEGG families of *Hypothenemus hampei* predicted proteins assigned by the BlastKOALA tool and grouped at the top level (a) and at the second level (b) of the KEGG BRITE hierarchy. Numbers indicate the percentage of all proteins in the dataset that received that classification.

The set of 19,222 predicted *H. hampei* proteins (https://genome.nyumc.org/CBB.htm) were functionally characterized in the KEGG PATHWAY Database (http://www.genome.jp/kegg/pathway.html) by the BlastKOALA sequence similarity tool (http://www.kegg.jp/blastkoala/) and by alignment to the PANTHER database of protein HMM models (http://pantherdb.org/panther/) with the PANTHER scoring tool (http://pantherdb.org/tools/hmmScoreForm.jsp). The KEGG BlastKOALA tool mapped 27% of the *H. hampei* predicted proteins (5,149 proteins) to KEGG ortholog groups, while PANTHER assigned 68% of the proteins to PANTHER families. In Fig. 1 we show the distribution of proteins at the top level (Fig. 1a) and second level (Fig. 1b) of the KEGG BRITE hierarchy.

### Gene Families Analyzed

The coffee berry borer spends most of its life cycle inside the coffee berry, where it consumes the seed. We therefore examined digestive enzyme composition, focusing on two digestive enzymes (amylases and mannanases), essential for catabolism of two representative coffee seed carbohydrates. Mannanases are discussed under horizontal gene transfer (below). Seeds are typically well defended against insect predators, so we also examined different detoxifying systems. While recent research indicates that coffee berry borer relies on bacteria in the midgut to detoxify caffeine (Ceja-Navarro *et al.*, submitted), we focused on genes potentially involved in caffeine detoxification as well as the detoxification of other plant allelochemicals and insecticides, such as the carboxylesterase gene family (CE), the cytochrome P450 gene family (CYP), the gluthathione S-transferase gene family (GST), the ATP-binding cassette transporter gene superfamily (ABC), and a gene (*Rdl*) that confers resistance to the insecticide dieldrin. In addition, we identified genes involved in pathogen defense, including homologs to antimicrobial genes reported in the *Drosophila* genome.

### Digestive Enzymes

The α-amylases are a family of simple carbohydrate (starch)-metabolizing enzymes common in animals, microorganisms, and plants^18^. Using the *T. castaneum* amylase gene (XP_969234) as query, we found exactly one orthologous match to assembled transcript C129694, with a BLAST e-value of 0.0 and 72% conserved amino acid matches. The *T. castaneum* amylase also matches by tblastn to exactly one genomic location on scaffold 1599, where the gene is predicted by GeneMark as evmscaffold1599.1. The gene is highly expressed, with an FPKM in our RNA-seq data of 52997. This transcript also shows homologous matches to α-amylase proteins from other Coleoptera, including the spruce bark beetle (*Ips typographus*) amylase B with a tBLASTn e-value of 6e^−142^ and 70% (predicted) amino acid identity; boll weevil (*Anthonomus grandis*) α-amylase (AAN77139) at e = 0.0 (55% amino acid identity); rice weevil (*Sitophilus oryzae*) at e = 0.0 (69% amino acid identity); corn rootworm (*Diabrotica virgifera virgifera*) α-amylase (AAF20998) at e = 0.0 (56% amino acid identity); azuki bean weevil (*Callosobruchus chinensis*) (BAD83655) at e = 0.0 (58% amino acid identity); Mexican bean weevil (*Zabrotes subfasciatus*) (AAF73435) at e = 0.0 (57% amino acid identity); and mustard beetle (*Phaedon cochleariae*) (CAA76926) at e = 0.0 (54% amino acid identity).

As expected, several additional digestive enzyme genes were present, however some unexpected ones were found that are likely important in the insect’s ability to use the seeds as a food source. Seeds typically have high levels of protease and amylase inhibitors, as well as lectins, designed to interfere with insect digestion and absorption. A wide variety of digestive proteinases were found, and included serine proteinases (e.g., chymotrypsin and trypsin), thiol proteinases (e.g., cathepsins), and metalloproteinases. While we cannot easily determine which of these are involved in digestion and which may have other roles until a gut transcriptome becomes available, it is clear that the insect has a potent suite of proteinases likely to be capable of dealing with plant defensive proteins. Most beetles have mainly thiol proteinases^19^, but the coffee berry borer appears to have a more extensive composition. Thus, it may be challenging to develop plant-produced protein based controls. Several articles have been published on the presence of amylases and proteases in the midgut of the coffee berry borer with the goal of expressing relevant inhibitors in coffee plants as a possible pest management strategy^1^. Nevertheless, the development of such a strategy remains a distant goal, complicated by the diversity of proteases present, as indicated by the present study. As would be expected, lipases are also present in the *H. hampei* genome.

The green coffee seed has high levels of hemicellulose (complex polysaccharides), pectins, and celluloses, which could serve as an important nutrient source if the complex matrix can be degraded^1^. Relevant digestive enzymes were identified, which are more typically associated with wood-feeding insects. The genome of the phloem feeding *D. ponderosae* contained polysaccharide endo-β-1,4 glucanases, endopolygalacturonases, glycoside hydrolases in family 48 (microcrystalline cellulases), rhamnogalacturonan lyases, and pectin methylesterases^5^. We found several of the same genes in the *H. hampei* genome, namely four glycosyl hydrolases in family 48, seven polygalacturonases, one pectin esterase, five rhamnogalacturonan lyases, and two beta glucanases. Additionally we found two mannanase and two xylanase genes that appear to be the result of horizontal gene transfer (see below). Although microbial associates of insects are reported to produce microcrystalline cellulases to the benefit of the associated insect^20^ their presence in the beetle host genome is not common, and has otherwise only been reported from the genome of a limited number of species^20^, including *Tribolium*^4^, which does not contain the other polysaccharide degrading enzymes^21^. The ability to degrade the hemicellulose/cellulose/pectin matrix could give the coffee berry borer a much more efficient use of its limited seed resource, and it has the greatest breadth of such genes so far reported for any beetle species. Developing strategies to interfere with these potentially important digestive enzymes may lead to as specific control mechanism for this insect. Cell walls are also typically lignified, which reduces their ability to be degraded. However, we did not find specific lignin degrading enzymes, such as laccases, although some of the peroxidases found in the genome may be capable of this function.

Genes for gustatory receptors were also found in the *H. hampei* genome, as were those for olfactory and pheromone receptors. Determination of these sequences allows for the potential development of feeding inhibitors, as well as attractants useful for monitoring, or attraction to insecticides of the free flying females.

### Detoxification of Plant Chemical Defenses

Seeds are typically well defended by plant secondary metabolites. The coffee seed is no exception, and contains high concentrations of a variety of chlorogenic acid derivatives^22^ as well as its principal chemical of human interest, caffeine. The chlorogenic acid derivatives can be toxic and in many cases contain appropriate chemical structures (such as aromatics with ortho- or para-hydroxyl or methoxy groups) that can act as substrates for phenoloxidases or peroxidases, which generate reactive oxygen species and toxic quinones^23^,^24^. Although tissue expression location has not been determined, genes to deal with reactive oxygen species, such as glutathione peroxidase, are present in the genome. Both cytochrome P450s and glutathione transferases (see below) would be capable of detoxifying these chlorogenates. Caffeine is a potent anti-insect compound found in plants^25^,^26^, and insects that feed on caffeine containing plants may have specialized detoxifying mechanisms.

### Caffeine detoxification

In another related study (Ceja-Navarro *et al.*, submitted), we have detected expression of methylxanthine N-demethylase genes (*ndm*) responsible for caffeine demethylation in *H. hampei*. However, we observed no homologs of this gene in the draft genome, suggesting that unlike mannanase (which has been reported to be present in the insect genome as a result of horizontal gene transfer from bacteria; discussed below), caffeine demethylation has not been laterally transferred to the genome and that this function resides in the bacterial community. This supports observations of elimination of this function through antibiotic treatment of coffee berry borers (Ceja-Navarro *et al.*, submitted).

### ATP-binding cassette transporter gene superfamily (ABC)

ABC transporters are membrane bound proteins that use adenosine triphosphate (ATP) binding and hydrolysis for the translocation of amino acids, lipids, sterols, sugars, peptides, xenobiotics, etc., across membranes, with the efflux of xenobiotics having been implicated in resistance to toxins (including insecticides)^27^,^28^.

**Figure 2:**
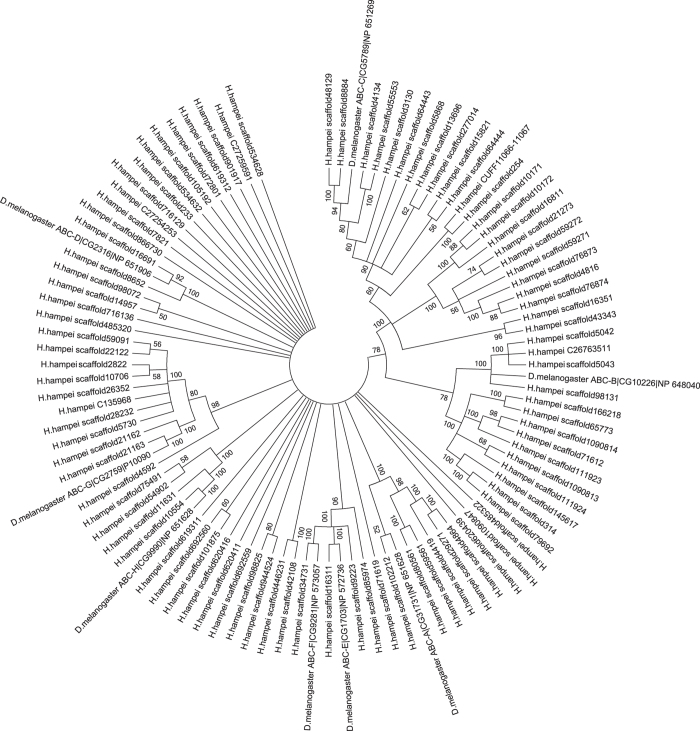
Maximum Likelihood tree (with 100 bootstraps, condensed at 50% bootstrap identity) of translated protein sequences of 95 putative ABC transporter genes identified in the draft genome of *Hypothenemus hampei* and proteins from *Drosophila melanogaster* representing the eight established ABC subfamilies (ABCA-ABCH). Tree built with MEGA6.

We searched for ABC transporters in the translated set of *H. hampei* predicted proteins using the hmmsearch program from the HMMER3 software package (http://hmmer.janelia.org)^29^ with the ABC_tran HMM model (PF00005) from the PFAM website (http://pfam.xfam.org)^30^. We found a total of 95 putative ABC transporters in the *H. hampei* genome (Fig. 2). We combined the protein sequences for these genes with annotated protein sequences from *D. melanogaster* for each of the eight established ABC transporter subfamilies (ABCA-ABCH). The sequences were multiple aligned with CLUSTALW and a maximum likelihood tree (with 100 bootstraps) was built using MEGA6 (http://www.megasoftware.net)^31^ (Fig. 2). We find the following distribution of subfamilies for the 95 predicted *H. hampei* ABC proteins ABC-A: six; ABC-B: 14; ABC-C: 24, ABC-D: two; ABC-E/F: four; ABC-G: 11, ABC-H: four; unidentified: 30. A total of 73 ABC genes were reported in *T. castaneum*^27^. The findings demonstrate a wide array of ABC transporter subfamilies is present in *H. hampei*; their specific functions await further study to determine their role in transporting toxic molecules out of cells.

### Carboxylesterase gene family (CE)

Carboxylesterase genes are involved in insecticide resistance and metabolism^32^,^33^, including several pyrethroid insecticides and the organophosphate insecticide malathion. *Apis mellifera* has 24 genes that are members of this gene family while *D. melanogaster* has 35^34^, *Aedes aegypti* has 49^35^, *Anopheles gambiae* has 51^32^, and *Tetranychus urticae* has 76^36^. The CE HMM model from the PFAM database (PF00135.23) was used to search the *H. hampei* predicted proteins using the hmmsearch program from the HMMER3 software package^29^. A total of 54 predicted proteins had significant matches to this conserved model (Table S3). In the *T. castaneum* genome assembly (NCBI version 4.0), there are 10 esterase genes that are highly similar to the *H. hampei* proteins (blastp e-values range from 0.0 to 1e^–144^), six of which form a tandem cluster on chromosome ChLG7, indicating a probable ancestral amplification of this gene. This means that the coffee berry borer has the appropriate gene resources to develop resistance to several insecticides.

### Cytochrome P450s gene family (CYP)

**Figure 3:**
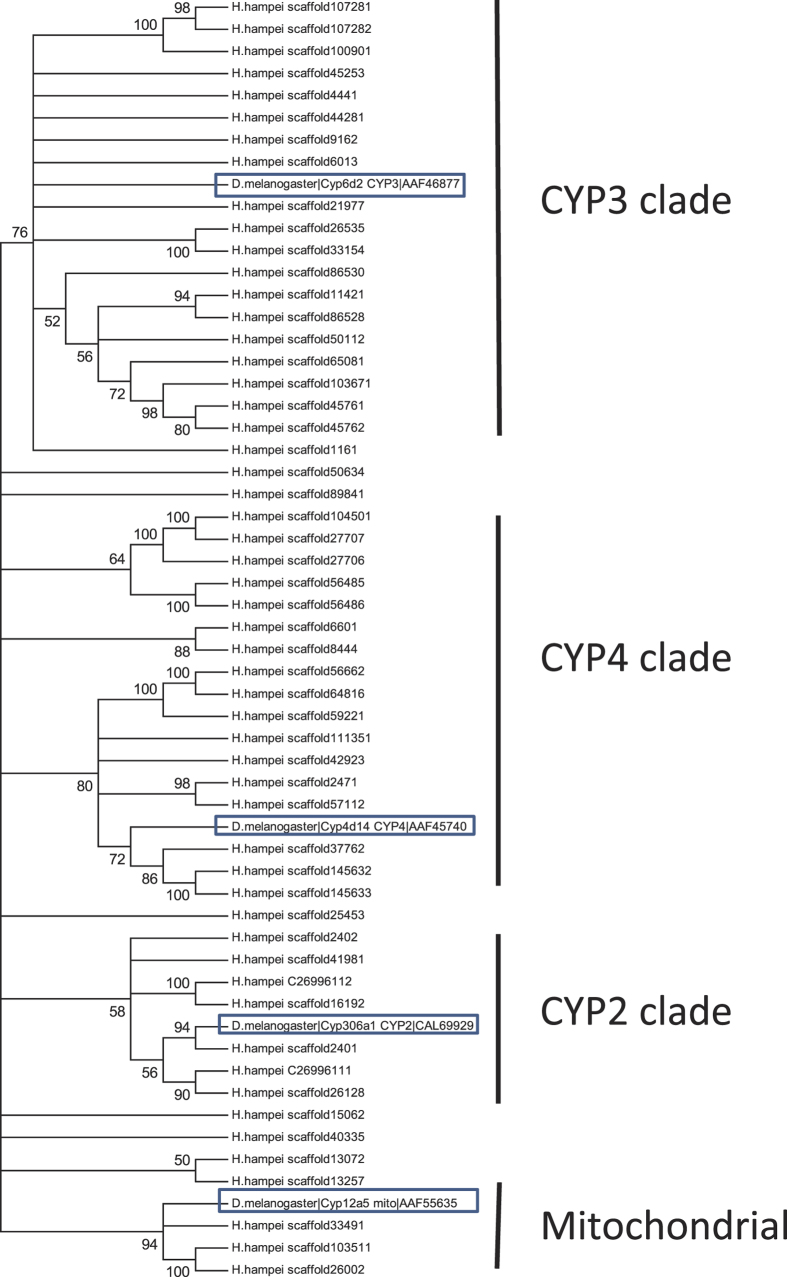
The 54 full-length cytochrome P450 genes predicted in the *Hypothenemus hampei* genome clustered with *Drosophila melanogaster* genes representing the four insect clades: CYP2, CYP3, CYP4, and mitochondrial. Predicted proteins in the cytochrome P450 family were identified by HMMER3 search with the Pfam CYP consensus model (PF00135.23). Evolutionary analyses were conducted using the Maximum Likelihood method in MEGA6. The percentage of replicate trees in which the associated taxa clustered together in the bootstrap test (100 replicates) are shown next to the branches. Branches with less than 50% bootstrap support are condensed.

Cytochrome P450s, present in all living systems^37^, are a superfamily of enzymes (monooxygenases) that, in insects, play a role in the synthesis and metabolism of hormones and pheromones and as well as in the breakdown (detoxification) of exogenous substrates such as plant allelochemicals and insecticides^37^. CYP families share >40% amino acid similarity, while subfamilies share >55%. Insect CYPs can be divided into four clades: mitochondrial, CYP2, CYP3, and CYP4^38^. The CYP HMM model from the PFAM database (PF00135.23) was used to search *H. hampei* predicted proteins using the hmmsearch program from the HMMER3 software package^29^. We identified 54 full-length CYP genes in the *H. hampei* draft genome (Table 2), with an additional 40 partial sequences. The 54 full-length *H. hampei* CYP genes were expressed at a wide range of different levels from FPKM 1e^6^ (the 148^th^ most highly expressed gene) down to FPKM 10. Expression level was not correlated with similarity to the CYP consensus HMM model. Of the 54 full-length CYP genes, three were mitochondrial, seven were CYP2, 20 CYP3, 17 CYP4, and seven undetermined, in contrast to *D. ponderosae*, whose 85 CYPs showed a different distribution (nine mitochondrial, seven CYP2, 47 CYP3, and 22 CYP4^9^). A phylogenetic reconstruction of Cyp450 genes from *H. hampei* with *Drosophila* as a reference shows families with gene loss and expansion (Fig. 3).

Cyp301a1 is a protein involved in cuticle formation and has been detected in all insect genomes^39^. An ortholog of the *D. melanogaster* Cyp301a1 was detected in scaffold2600.2 of the draft genome (e = 0.0, amino acid identities = 331/480 (69%), positives = 397/480 (83%), gaps = 3/480 (1%)).

The proteins coded by some of these CYP genes are likely responsible for detoxifying the chlorogenic acids present in the seed. Again, some of these could potentially contribute to insecticide resistance. Insect P450s involved in the detoxification of a wide range of xenobiotics have nonpolar residues in the hydrophilic cage surrounding the aromatic network responsible for substrate positioning, and nonaromatic residues in regions SRS2, SRS5 and SRS6^40^. An epoxide hydrolase gene was also found in the genome. Epoxides can be generated by P450s, and if a molecule such as aflatoxin (which has been reported in coffee seeds^41^) is present, will produce a compound that will intercalate into DNA and result in toxic DNA effects^42^. Epoxide hydrolases will break this epoxide and prevent such adverse reactions on the DNA^42^.

Determining the substrate capabilities of the P450s may help guide choices for insecticides for control and selecting those that are less likely to develop resistance due to an absence of capable P450s. For example, based on studies reported above, it does not appear that the *H. hampei* has N-demethylase capability of its own. Thus, using contact insecticides that contain N-methyl groups attached to aromatic molecules, compared to other structural moieties, may be preferable for longer lifespan controls.

### Glutathione S-transferase gene family (GST)

**Figure 4:**
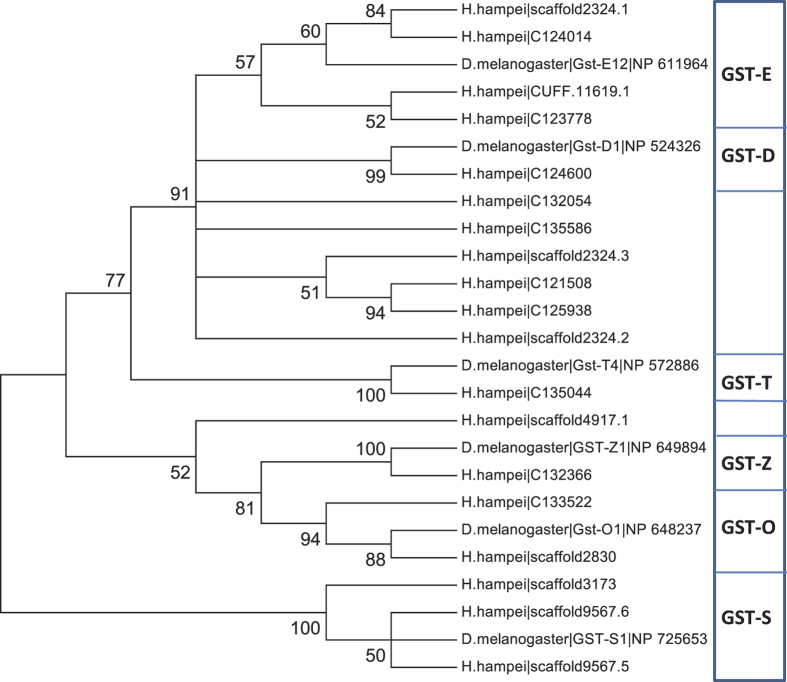
Maximum likelihood tree of 19 predicted *Hypothenemus hampei* glutathione S-transferase proteins with selected *Drosophila melanogaster* proteins representative of the six characterized GST classes. *Hypothenemus hampei* proteins were identified by significant hmmsearch similarity scores using the Pfam GST-C (PF00043) and GST-N (PF02798) HMM models. The tree was constructed with MEGA 6 using the JTT matrix-based model, with 100 bootstraps. The percentage of trees in which the associated taxa clustered together is shown next to the branches. Branches with less than 50% bootstrap support are condensed. Evolutionary analyses were conducted in MEGA6.

The GST gene family is present in all aerobic organisms and is involved in the mediation of oxidative stress responses, hormone biosynthesis, and in the detoxification of plant allelochemicals and insecticides^43^. Six GST classes have been identified in insects, with members within a class sharing >40% amino acid identity^43^,^44^. Using a combination of BLAST searches with annotated GST genes from *Drosophila* and HMM searches with PFAM GST families, 19 predicted GST genes were identified in the *H. hampei* genome and transcriptome, distributed throughout all six classes as follows: one Delta, four Epsilon, two Omega, three Sigma, one Theta, and one Zeta (Fig. 4). Five of the *H. hampei* genes are similar to both Epsilon and Delta classes (44% amino acid identity to both *Drosophila* GST D and E genes) but cannot be clearly distinguished between them. Homologs of these unclassified genes (63% amino acid identity) in *D. ponderosae* are annotated as hypothetical proteins. At least some of these GSTs are likely involved in detoxifying the chlorogenates in the seed and could contribute to detoxification of insecticides as well. Delta class GSTs are associated with resistance to DDT and are involved in resistance to some organophosphate insecticides, while omega forms can readily act on aromatic substrates^45^, so both classes are likely to be involved in detoxification. The Zeta class GSTs have a very small active site; the Sigma class are involved in prostaglandin synthesis, and the Theta class are inactive toward chlorinated aromatics^45^, so are less likely to be involved in detoxification. Thus, *H. hampei* has GSTs likely to be involved in plant defensive compound and insecticide detoxification.

### Resistance to dieldrin gene (Rdl)

The broad-spectrum chlorinated cyclodiene insecticide endosulfan (C_9_H_6_Cl_6_O_3_S) has been used in many countries in an attempt to control *H. hampei*, but due to its human and environmental toxicity, the insecticide has been banned in at least 70 countries^1^. Mutations (Ala-302 → Ser) in the *Rdl* gene (resistance to dieldrin), which encodes the γ-aminobutyric acid subtype A (GABAA) receptor, are responsible for the resistance to the cyclodiene group insecticides in many insects, including *A. aegypti, D. melanogaster, Musca domestica, Periplaneta americana, and T. castaneum*^46^. A genomic sequence of the *H. hampei* Rdl gene from an endosulfan resistant strain was deposited in GenBank (AF037324)^47^ and contains the Ala → Ser resistance mutation at an equivalent position. *Hypothenemus hampei* resistance to endosulfan has been reported in New Caledonia^48^ and molecular detection of the cyclodiene resistance gene *Rdl* has been demonstrated in insects from New Caledonia^47^,^49^ and Colombia^50^.

The *H. hampei* Rdl gene is located on scaffold 355 in the draft genome. The gene predicted by GenMark/EVM shows 98% nucleic acid identity to GenBank accession AF037324 over its entire length. However, very few RNA-seq reads align to this locus and the transcript is not fully reconstructed using SOAPdenovo-Trans or Cufflinks. The translation of the predicted gene aligns with the protein sequence of the *T. castaneum* GABA receptor isoform X1 (BLAST e-value 0.0, 77% amino acid identity) and shows a serine at an equivalent site to the resistance mutation that has been reported in endosulfan resistant flies, beetles and other insects. The insects used to establish the laboratory colony used in the present study originated in Colombia and have been under continuous rearing in the laboratory for ca. 10 years and hundreds of generations, indicating that even though there is no longer selective pressure, the resistant form of the gene has been maintained.

### Pathogen Defense Genes

Insects have a complex pathogen defense system that consists of pathogen recognition, humoral responses (antimicrobial peptides), cellular responses, melanization, and intracellular defenses (RNAi)^51^. A variety of lectin genes potentially involved in pathogen recognition were detected in the *H. hampei* draft genome. These include lectin C, mannose and galactose binding lectins, as well as peptidoglycan receptor proteins (see below). The regulatory protein gene Spätzle was also present. A prophenoloxidase gene with 77% homology to two *Tribolium* prophenoloxidases (XM_008196766.1 and NM_001039433) was present in the draft genome, although it was annotated during our analysis as Hemocyanin_C , 6586. Several serpin genes, which can regulate the phenoloxidase activity in insects and shrimps^52^, do occur in the draft genome. Two Dicer genes, which are involved in the RNA interference (RNAi) process, were also present in the draft genome.

### Homologs to Drosophila antimicrobial peptides and related genes

We identified additional putative antimicrobial-related genes by BLAST using the set of all *D. melanogaster* genes annotated with the following Gene Ontology (GO) database terms (http://amigo.geneontology.org/amigo/landing): antimicrobial humoral response (GO:0019730), antibacterial humoral response (GO:0019731), or defense response to bacterium (GO:0042742). A total of 175 genes were identified including 18 w, akirin, andropin, attacin, cecropin, defensin, diptericin, drosomycin, listericin, lysozyme, etc. Of these 175 *Drosophila* antimicrobial genes, we find that 120 of them match predicted *H. hampei* proteins with BLAST significance scores of 5e^−10^ or lower. For the reciprocal search, a total of 749 predicted *H. hampei* proteins match the set of *Drosophila* anti-microbial genes with BLAST significance scores of 5e^−10^ or lower, showing that these gene families are conserved and have abundant members in the *H. hampei* genome. A search of the *T. castaneum* proteome (21,468 GenBank proteins) with the same set of 175 *D. melanogaster* antimicrobial proteins finds 1,075 matches with BLAST e-values of 5e^−10^ or lower.

**Figure 5:**
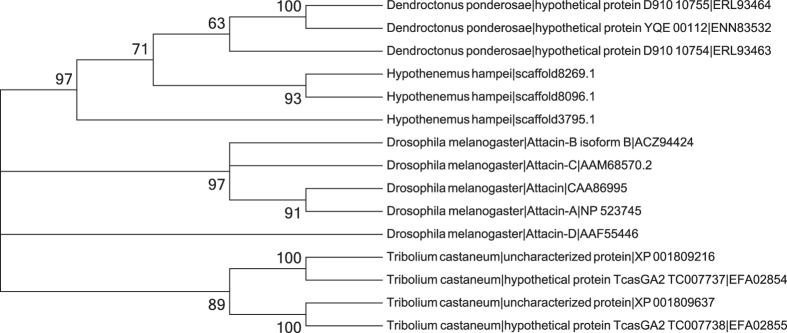
Maximum likelihood tree of attacin protein sequences from *Dendroctonus ponderosae*, *Drosophila melanogaster*, and *Tribolium castaneum*, and three predicted proteins from *Hypothenemus hampei*. Tree was constructed with MEGA 6 using the JTT matrix-based model, with 100 bootstraps. The percentage of trees in which the associated taxa clustered together is shown next to the branches. Branches with less than 50% bootstrap support are condensed. Evolutionary analyses were conducted in MEGA6.

*Drosophila melanogaster* has five attacin proteins (attacin, attacin-A, -B, -C, and -D). Four uncharacterized homologs are found in the genome of *T. castaneum* and three in the genome of *D. ponderosae*. Three significantly similar *H. hampei* predicted proteins (evm.model.scaffold8269.1, evm.model.scaffold8096.1, evm.model.scaffold3795.1; Fig. 5) were found by BLAST searches with this set of query sequences. All three *H. hampei* proteins were highly expressed with FKPM values of 12524, 12025, and 2098 respectively. The same three proteins were found by hmmsearch with PFAM HMM model PF03769 (attacin-C). Phylogenetic analysis does not show a clear orthology relationship among the attacin proteins from these species, rather the sequences from each species cluster together.

*Tribolium castaneum* has an annotated cecropin gene (NP_001164146), but no significant similarity to this gene was found by blastp to *H. hampei* predicted proteins or by tblastn to the *H. hampei* transcript assemblies or to the *H. hampei* draft genome sequence. No PFAM matches to the cecropin HMM model (PF00272) were found for *H. hampei* predicted proteins.

Coleoptericin is a glycine rich antimicrobial protein found in many Coleopteran species. Homologs have been identified in the draft genomes of *T. castaneum* (XP_008199794) and *D. ponderosae* (ERL87200). All of these proteins have significant blastp matches to *H. hampei* predicted protein evm.model.scaffold348.1 (7e^−37^, 70% amino acid identity). No PFAM matches to the coleoptericin HMM model (PF06286) were found for *H. hampei* predicted proteins.

A blastp search of *H. hampei* predicted proteins with the defensin peptide sequence from the Japanese rhinoceros beetle *Allomyrina dichotoma* (gb|AAB36306) produced one significant match (evm.scaffold2042.7), which is highly expressed (FPKM 10520). A hmmsearch with PFAM HMM model PF01097 (Defensin_2) also matches only to evm.model.scaffold2042.7, whose predicted protein has significant blastp similarity to defensin from several insect species with 50–60% amino acid identity, including holotricin (*T. castaneum*), coprisin (*Copris tripartitus*), tenecin (*Tenebrio molitor*), and phormin (*Protophormia terraenovae*).

Drosomycin is found primarily in flies. The *Drosophila* drosomycin protein (AAF47767) was used as a query, but no significant similarity was found by blastp to *H. hampei* predicted proteins or by tblastn to the *H. hampei* transcript assemblies or to the *H. hampei* draft genome sequence. Similarly, moricins and gloverins have been found only in Lepidoptera (e.g., BAB13508 and CAL25129). BLAST searches of *H. hampei* predicted proteins, assembled transcripts, and draft genome sequence did not yield any significant similarities to these proteins. Proline rich antimicrobial proteins such as lebocin (NP_001037468) and drosocin (CAA79936) are found in Diptera, Hymenoptera, Hemiptera, and Lepidoptera. No homologs of these genes have been reported in Coleoptera. BLAST searches with sequences of drosocin and lebocin did not find any significant matches in the *H. hampei* predicted proteins, assembled transcripts, or draft genome sequence.

The detection of antimicrobial peptides genes reveals that the coffee berry borer possesses a wide array of mechanisms that could be used for antimicrobial defence. These peptides have been reported to have activity against both fungi and bacteria^53^.

### Horizontal Gene Transfer (HGT)

A mannanase gene (GenBank ID ACU52527) similar to a *Bacillus circulans* protein was identified from a *H. hampei* cDNA^54^ and a xylanase (GenBank ID ADN94682) highly similar to *Streptomyces bingchenggensis* was isolated from a *H. hampei* genomic clone^55^. All predicted *H. hampei* proteins from the draft genome were screened for HGT candidates by identifying proteins with more significant BLAST e-values to a set of bacterial proteins (UniRef50 bacterial proteins) than to a set of arthropod proteins (UniRef50 arthropod proteins). To remove genes originating from contaminating bacterial DNA in the *H. hampei* genomic library, only gene models with >10% of exon bases covered by aligned RNA-seq reads were considered as HGT candidates.

A total of 10 HGT candidate genes were identified with evidence for expression, integration into the *H. hampei* genome, and phylogenetic evidence that the sequences are more closely related to bacterial rather than eukaryotic genes (Table 3). Two copies of endo-1,4-beta-xylanase genes with high similarity to *Streptomyces* sp. were found on two different draft genome contigs (BLAST e-value ~1e^−107^, 46–56% amino acid identity). These predicted proteins have 89% and 86% amino acid identity respectively to the xylanase gene reported by Padilla-Hurtado *et al.*^55^, but only 75% amino acid identity to each other. These predicted *H. hampei* genes are highly expressed (FPKM 1.5e^6^ and 3.1e^5^, based on Cufflinks analysis of aligned RNA-seq reads), contain introns, and are on contigs that contain other predicted *H. hampei* genes. A similar endo-1,4-beta-xylanase (BLAST e-value 8e^−40^) was identified in the gut microbiota of the termite *Globitermes brachycerastes*, but is incorrectly annotated in GenBank ID 4HU8_A as of arthropod origin^56^. Xylanases present in the mustard leaf beetle, *Phaedon cochleariae* (Chrysomelidae) also appear to have been acquired from bacteria^57^. Polygalacturonase genes apparently acquired from bacterial or fungal sources have been reported for some species of cerambycids, chrysomelids, and curculionids^58^.

**Figure 6:**
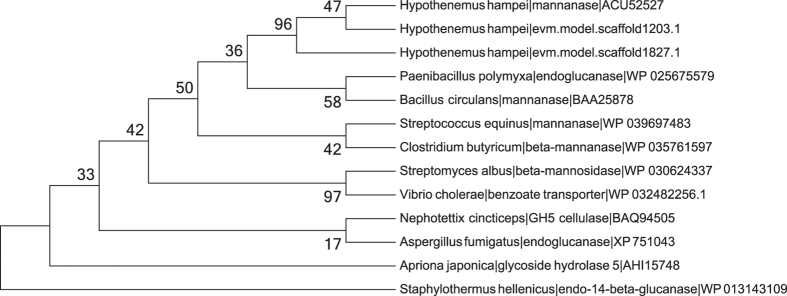
Maximum likelihood tree including the protein sequence of the previously reported *Hypothenemus hampei* mannanase gene (GenBank ID ACU52527), two highly similar predicted proteins from the *H. hampei* draft genome (scaffold1203.1 and scaffold1827.1) with homologs from eubacteria, fungal, and insect proteins. The beta-glucanase protein from the archaebacteria *Staphylothermus hellenicus* is included as an outgroup. The *H. hampei* proteins clearly cluster with the eubacterial proteins. The percentage of replicate trees in which the associated taxa clustered together in the bootstrap test (100 replicates) are shown next to the branches. Evolutionary analyses were conducted using MEGA6.

Two copies of mannanase genes of possible bacterial origin were found on two different contigs of the draft genome (Table 3; Fig. 6). The previously reported HhMAN1 gene (GenBank ID GQ375156^54^) matches scaffold1203.1 with 100% DNA sequence identity over a region of 5 kb, including a predicted transcript, which is highly expressed (FPKM 4.3e^6^). A second predicted gene on scaffold1827.1 was identified with similarity to mannanase from *Bacillus* (BLAST e-value 2e^−30^, ~59% amino acid identity). This gene has ~94% DNA sequence identity to GQ375156 over a much smaller region of 562 bases and 91% amino acid identity to the HhMAN1 protein (GenBank ID ACU52526) over the coding sequence. This second mannanase gene is expressed at FPKM 3.7e^4^.

Predicted gene scaffold3436.2 has strong similarity to hydrolase proteins from *Streptomyces* (BLAST e-value 0.0, 43% amino acid identity) and no significant similarity to any arthropod genes (best BLAST value >2.0) (Table 3). The gene is highly expressed (FPKM 1.5e^4^) and is located on a scaffold with other predicted genes that have homology with insect proteins.

**Figure 7:**
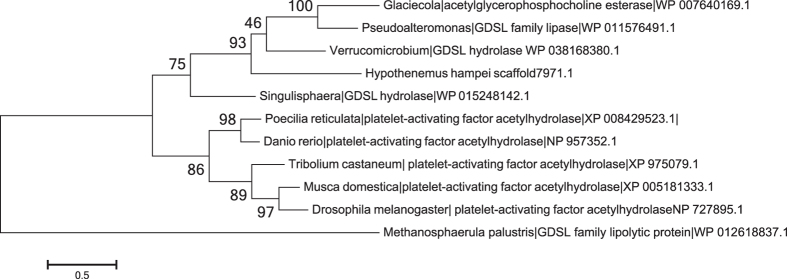
Maximum likelihood tree of the translated protein sequence of the predicted *Hypothenemus hampei* GDSL lipase/acylhydrolase gene (scaffold7971.1) with homologs from eubacteria, animal, and insect proteins. The GDSL family lipolytic protein from the archaebacteria *Methanosphaerula palustris* is included as an outgroup. The *H. hampei* protein clearly clusters with the eubacterial proteins. The percentage of replicate trees in which the associated taxa clustered together in the bootstrap test (100 replicates) are shown next to the branches. Evolutionary analyses were conducted using MEGA6.

A predicted *H. hampei* gene (scaffold7971.1) has stronger similarity to a Proteobacteria lipase/acylhydrolase (BLAST e-value 1e^−37^, 37% amino acid identity) than to its closest arthropod match, platelet activating factor acetylhydrolase (BLAST e-value 1e^−21^, 32% amino acid identity) (Table 3). A maximum likelihood tree including a sample of protein sequences from KEGG orthology group K01137 (EC:3.1.6.14) clearly shows that the *H. hampei* gene is much more closely related to bacterial than to eukaryotic genes (Fig. 7). Aligned RNA-seq reads show that the gene is highly expressed (FPKM 4.9e^6^) and has a spliced intron.

A predicted gene on scaffold 537.2 has homology to hypothetical proteins from *Rickettsia* (BLAST e-value 2e^−31^) (Table 3), but is supported by only a few RNA-seq reads and is not included among transcript models created by Cufflinks or SOAPdenovo-Trans.

Predicted protein scaffold 3881.7 is similar to *Wolbachia* regulatory protein RepA (BLAST e-value 9e^−34^) (Table 3), but has lesser levels of similarity with other bacteria such as *Ralstonia, Candidatus, Xanthomonas*, as well as gp5 originating from bacteriophage WO. It has no significant similarity to any arthropod genes. It is expressed at a moderate level (FPKM 3.2e^4^).

Predicted protein scaffold1344.1is similar to *Citrobacter* Ig-like protein (BLAST e-value 2e^−23^) invasin (Table 3), with no significant similarity to any arthropod genes. It is expressed at FPKM 1.9e^4^.

Predicted protein C2660673.1 has strong similarity to permease from *Lactobacillus* and other Firmicutes (BLAST e-value 2e^−53^, 66% amino acid identity) (Table 3). The closest similarity to an arthropod gene is a BLAST e-value of 6e^−22^ with a short 88 amino acid segment of hypothetical protein from the tick *Ixodes scapularis*. The gene is on a small contig (1.1 kb) with moderate RNA-seq coverage (FPKM 2.3e^3^), gene model prediction by Cufflinks, SOAPdenovo-Trans, and PASA/EvidenceModeler, but no evidence of splicing.

The 10 HGT candidate genes in the *H. hampei* genome confirm the close relationship the insect has had with bacterial populations. For example, Ceja-Navarro *et al.* (submitted) identified several bacteria isolated from the alimentary canal that are able to subsist on caffeine as the sole source of carbon and nitrogen, and demonstrated that caffeine detoxification by the coffee berry borer is meditated by its microbiota.

## Conclusions

The draft *H. hampei* genome is the third Coleoptera genome and the second bark beetle genome to have been analyzed. The most important findings in the coffee berry borer genome include (1) evidence for 10 cases of horizontal gene transfer from diverse bacteria; (2) paralogous expansion of antimicrobial peptides; (3) presence of a broad range of enzymes capable of degrading complex polysaccharides; and (4) the presence of a gene (*Rdl*) that confers resistance to a cyclodiene insecticide. The genome should be a useful tool for other researchers studying Coleoptera, and in particular, to those studying economically important bark beetles. The genome has also provided further understanding as to how *H. hampei* is able to successfully exploit its food resource and defend against pathogens. The information provided also can help guide the development of management strategies related to use of insecticides, biological control agents, attractants and plant resistance mechanisms that could be developed by breeding or genetic engineering.

## Methods

### Nucleic Acid Extraction and Purification

Female coffee berry borers had been continuously reared on a meridic diet in the laboratory^59^ for ca. 10 years. Ten batches of 100 female insects were placed in microcentrifuge tubes containing 1 ml of RNA*later*^*®*^ (Ambion, Grand Island, NY, USA) and incubated overnight at 4°C. After the initial incubation, the RNA*later*^*®*^ was removed, and the insects’ cuticle was sterilized by treating each batch with four sequential washing steps consisting of 500 μl of DEPC-treated water, commercial bleach, absolute ethanol and DEPC-water. At each step, the sample was vigorously shaken for ca. 30 sec. The washing of the samples was done twice. The insects from each batch were then transferred to a Lysing Matrix E tube (MP Biomedicals, Santa Clara, CA, USA) containing 500 μl of lysis solution (2% CTAB in 0.5 M NaCl). The samples were bead-beaten in a FastPrep Instrument (MP Biomedicals) at 4 m/s for 30 seconds. Lysozyme buffer (180 μl; 20 mM Tris-HCl (pH 8.0), 2mM EDTA, 1.2% Triton X-100) containing 10 mg/ml of lysozyme was added to each tube and the mixture incubated at 37 °C for 30 min. Five μl of proteinase K (Ambion, Grand Island, NY, USA) was added to the tubes, mixed by tapping and incubated at 56°C for 30 minutes. After the proteinase K lysis, 500 μl of phenol:chloroform:isoamyl alcohol (25:24:1) was added to each tube, and the samples bead-beaten in the FastPrep instrument as before. The samples were centrifuged at 10,000 × g for 1 min at 4 °C and the supernatants transferred to a MaXtract high density tube (Qiagen, Germantown, MD, USA) containing 500 μl of chloroform:isoamyl alcohol (24:1). The samples were centrifuged 10,000 × g for 1 min at 4 °C, and the supernatants transferred to a microcentrifuge tube containing 1 ml of isopropanol and 1 μl of linear acrylamide (Ambion, Grand Island, NY, USA). The DNA/RNA mixture was precipitated by incubating for 10 min at room temperature and centrifuged at 10,000 × g for 5 min at 4 °C, and the isopropanol removed. The obtained pellet was washed with 70% ethanol and centrifuged at 10,000 × g for 1 min at 4 °C. The ethanol was completely removed, and the pellet dissolved in DEPC-treated water. The DNA and RNA were purified and separated with the AllPrep DNA/RNA kit (Qiagen) following the manufacturers protocol that was modified to treat the samples with either DNase or RNase (Ambion) for 5 min before the washing steps. The DNA and RNA concentration was determined with the use of a Qubit fluorometer (Qiagen) and the RNA quality assessed by bioanalyzer. The extracted DNA and RNA were finally pooled to a final concentration of 10 μg for genomic and transcriptomic library preparation.

### Protocol for Coffee Berry Borer noPCR Libraries

Genomic DNA from whole coffee berry borers was sheared to 350 and 550 bp sized fragments. To remove any amplification bias at the PCR step of the library preparation, noPCR libraries were prepared from coffee berry borer DNA using Illumina TruSeq DNA PCR-Free LT Sample Preparation Kit according to manufacturer’s instructions. Briefly, 1–2 ug of genomic DNA was fragmented using Covaris shearing with prescribed settings for 350 or 550 bp insert sizes. The fragmented DNA was cleaned, end-repaired, and selected for appropriate insert sizes (350 bp and 550 bp) using different ratios of Sample Purification Beads. This was followed by 3′ end adenylation and adapter ligation. Libraries were sequenced in Illumina Hiseq2000 with 100 bp paired-end reads.

### Protocol for Coffee Berry Borer ssRNAseq Library

Strand-specific mRNA sequencing libraries were prepared from total RNA of coffee berry borer using TruSeq Stranded mRNA Sample Prep Kit LT (Illumina) according to manufacturer’s instructions. Briefly, polyA + mRNA was purified from total RNA using oligo(dT) Dynabeads (Life Technologies) selection. First strand cDNA was synthesized using randomly primed oligos followed by second strand synthesis where dUTPs were incorporated to achieve strand-specificity. The cDNA was adapter-ligated and the libraries amplified by PCR. Libraries were sequenced in Illumina Hiseq2000 with paired-end 100 bp read chemistry.

### Genome Assembly

The genomic reads were cleaned with Trimmomatic^60^ to trim low quality reads (including leading or trailing sequences with the quality less than 20, any 4-base window with the quality less than 25) and Illumina TruSeq 3 adapter artifacts, filtered for bacterial contamination by BLAST searching.

After quality filtering, a total of 62 Gbp of sequence data (36 Gbp from the 350 bp insert library and 26 Gbp from the 550 bp insert library) was used for genome assembly. With an estimated genome size of 200 Mb, this corresponds to an average depth of coverage of 180-fold. Sequence reads were assembled using SOAPdenovo2 to form contigs and scaffolds resulting in 156 Mb in contigs and 163 Mb in scaffolds (see Table 1 and Table S1 for assembly statistics).

### Gene Prediction

*Ab initio* gene prediction on the assembled genomic contigs and scaffolds was made with GeneMark.hmm-ET^12^. Potentially protein-coding regions on the DNA assemblies were identified by tBLASTn similarity with a set of non-redundant conserved proteins from the UniProt Knowledgebase (UniRef90^13^).

RNA-seq data was used to *de novo* assemble transcripts using the SOAPdenovo-Trans software^8^, producing an overall count of 54,068 transcript assemblies. The transcript assemblies produced by SOAPdenovo-Trans were mapped to the genome with BLAT v35^14^ and then analyzed by PASA to optimize intron splicing sites, producing genome aligned spliced transcripts. EVidenceModeler^11^ was used to combine the PASA transcripts with GeneMark predicted genes, and with the genomic regions showing BLAST similarity to known proteins, which produced a final set of 20,301 gene models and translated predicted proteins.

An additional set of “genome guided” predicted transcripts was produced from a combination of RNA-seq and genomic data using the TopHat/Cufflinks software package. RNA-seq data were aligned to the genomic contigs using TopHat2^9^, then Cufflinks2^10^ was used for transcript assembly, yielding a set of 18,797 predicted transcripts.

The transcripts produced by Cufflinks and the predicted protein sequences produced by GeneMark/EVM were annotated by BLAST comparison with the NCBI non-redundant protein database. Gene functions (Fig. 1) were inferred from Genome Ontology information of these matching proteins using Blast2GO software.

### Data Availability

The assembled genomic contigs and predicted proteins for the *H. hampei* genome are available on a GBrowse server hosted at NYU Medical Center: https://genome.med.nyu.edu/gb2/gbrowse/hham. Tracks are available for the DNA contigs, *de novo* assembled RNA transcripts, Cufflinks predicted transcripts, and GeneMark/EVidenceModeler predicted gene models as well as regions of homology to UniProt proteins.

## Additional Information

**How to cite this article**: Vega, F. E. *et al.* Draft genome of the most devastating insect pest of coffee worldwide: the coffee berry borer, *Hypothenemus hampei*. *Sci. Rep.* **5**, 12525; doi: 10.1038/srep12525 (2015).

## Supplementary Material

Supplementary Information

## Figures and Tables

**Table 1:** *Hypothenemus hampei* genomic DNA (SOAPdenovo2) and RNA-seq (SOAPdenovo-Trans) assembly statistics.

**DNA**	
N50 scaffolds, bp	44,715
Genome size (including N), Mb	162,950,840
Genome size (without N), Mb	156,695,323
Scaffold number	86,848
GC content	32.46%
N50 contigs, bp	10,499
Longest scaffold, bp	440,081
Gene models (predicted genes)	19,222
**RNA**	
N50	1,638
Size (including N)	28,722,952
Size (without N)	28,327,000
GC content	35.88%

**Table 2:** A total of 54 predicted cytochrome P450 genes were identified in the *Hypothenemus hampei* draft genome.

***H. hampei* protein**	**FPKM**	**HMMERe-value**	**CYPclade**	**CUFFLINKStranscript**
evmmodelscaffold6481.6	1004190	1.70E-98	CYP4	CUFF.12736.1
evmmodelscaffold2770.7	375522	5.90E-80	CYP4	C125194
evmmodelscaffold865.28	240521	1.20E-69	CYP3	CUFF.14635.1
evmmodelscaffold2197.7	183475	2.70E-84		CUFF.4464.1
evmmodelscaffold1456.32	174361	1.50E-172	CYP4	CUFF.3616
evmmodelscaffold4033.5	147147	4.10E-54	Mito	CUFF.9032.1
evmmodelscaffold1325.7	101273	2.10E-33	Mito	CUFF.2068.1
evmmodelscaffold2653.5	99649	2.40E-71	CYP3	CUFF.5808.1
evmmodelscaffold1456.33	65672	4.00E-108	CYP4	CUFF.3617
evmmodelscaffold4525.3	65455	4.10E-83	CYP3	CUFF.9829
evmmodelscaffold10450.1	60925	1.20E-64	CYP4	CUTT.985.2
evmmodelscaffold571.12	53606	1.70E-104	CYP4	CUFF.11699.1
evmmodelscaffold1307.2	49167	5.70E-45	Mito	—
evmmodelscaffold844.4	46252	6.90E-60		CUFF.14420.1
evmmodelscaffold3349.1	45619	2.50E-64	Mito	CUFF.7694.1
evmmodelscaffold4576.1	40606	5.20E-65	CYP3	CUFF.9942.1
evmmodelscaffold1506.2	40106	3.50E-79	Mito	CUFF.2636.1
evmmodelscaffold4576.2	37291	1.30E-66	CYP3	CUFF.9937.1
evmmodelscaffold247.1	36092	1.50E-88	CYP4	CUFF.5327.1
evmmodelscaffold10728.2	35742	6.00E-102	CYP3	CUFF.1158.1
evmmodelscaffold916.2	27657	1.50E-100	CYP3	CUFF15114.1
evmmodelscaffold4428.1	20286	2.00E-98	CYP3	CUFF.9689.1
evmmodelscaffold8984.1	20186	5.50E-46	CYP4	CUFF.14931.1
evmmodelscaffold4198.1	19820	4.30E-65	CYP2	CUFF.9256.1
evmmodelscaffold4292.3	18337	2.70E-107	CYP4	CUFF.10885
evmmodelscaffold1142.1	16123	2.30E-66		C132918
evmmodelscaffold240.1	14007	5.20E-92	CYP2	CUFF.5111.1
evmmodelscaffold3776.2	12574	9.00E-101	CYP4	CUFF.8596
evmmodelscaffold5922.1	11959	4.80E-98	CYP4	CUFF.11953.1
evmmodelscaffold2612.8	11249	3.60E-68	CYP2	—
evmmodelscaffold5063.4	10986	3.30E-39		CUFF.10760.1
evmmodelscaffold10090.1	10659	1.40E-82	CYP3	CUFF.708.1
evmmodelscaffold240.2	10581	1.00E-110	CYP2	CUFF.5112.1
evmmodelscaffold559.1	10526	2.10E-10		CUFF.11505
evmmodelscaffold865.30	10475	5.30E-85	CYP3	CUFF.14635.2
evmmodelscaffold10367.1	10240	3.40E-61	CYP3	CUFF.952.1
evmmodelscaffold2770.6	7772	8.10E-87	CYP4	CUFF.6104.2
evmmodelscaffold9450.1	6295	4.00E-50		CUFF.15342.1
evmmodelscaffold3315.4	5399	6.40E-76	CYP3	CUFF.7475.1
evmmodelscaffold5648.6	4430	5.50E-79	CYP4	CUFF.1157.1
evmmodelscaffold1619.2	3247	2.40E-64	CYP2	CUFF.2900.1
evmmodelscaffold6508.1	3198	4.40E-87		CUFF.12657
evmmodelscaffold2545.3	3128	6.50E-32		CUFF.5506.1
evmmodelscaffold116.1	2531	4.30E-82	CYP3	CUFF.1594.1
evmmodelC2699611.1	2099	4.90E-14	CYP3	CUFF.639.1
evmmodelC2699611.2	2099	4.40E-68	CYP2	—
evmmodelscaffold5011.2	2098	1.20E-54		CUFF10656.1
evmmodelscaffold444.1	1850	1.90E-77	CYP3	CUFF.9710.1
evmmodelscaffold10728.1	1532	3.30E-106	CYP3	CUFF.1152.1
evmmodelscaffold2600.2	1327	4.00E-150	Mito	CUFF.5658.1
evmmodelscaffold5648.5	100	2.20E-99	CYP4	—
evmmodel10351.1	20	4.10E-13	Mito	—
evmmodelscaffold5666.2	10	4.70E-95	CYP4	—
evmmodelscaffold601.3	2	5.40E-80	CYP3	—

Gene expression is shown as FPKM values generated by Cufflinks. Cytochrome protein function is inferred by motif analysis of translated PASA/EVM predicted gene coding regions with HMMER hmmsearch (v.3.0) using the consensus cytochrome HMM model from PFAM (PF00067.17). Cytochrome clade was inferred by phylogenetic clustering with reference genes from *D. melanogaster* by minimum evolution with MEGA6.

**Table 3:** Ten candidate horizontally transferred genes detected in *Hypothenemus hampei*.

**Gene ID**	**RNA-seq expression (FPKM)**	**Bacterial BLAST match**	**e-value**	**Closest arthropod BLAST match**	**e-value**	**Predicted gene function**
scaffold769.3	1.5e^6^	*Streptomyces*	1e^−107^	*Musca domestica* (Diptera)	0.016	Xylanase
scaffold2902.1	3.1e^5^	*Streptomyces*	1e^−107^	*Stegodyphus* (Araneae)	1.0	Xylanase
scaffold1203.1	4.3e^6^	*Bacillus*	2e^−30^	*Anopheles darling* (Diptera)	1.1	Mannanase
scaffold1827.1	3.7e^4^	*Bacillus*	1e^−57^	*Cerapachys biroi* (Hymenoptera)	3e^−7^	Mannanase
scaffold3436.2	1.5e^4^	*Streptomyces*	0.0	*Nilaparvata lugens* (Homoptera)	2.2	Hydrolase
scaffold7971.1	4.9e^6^	*Protobacteria*	1e^−37^	*Tribolium castaneum* (Coleoptera)	1e^−21^	Lipase/acyl-hydrolase
scaffold537.2	*—*	*Rickettsia*	2e^−31^	none	—	Hypothetical
scaffold3881.7	3.2e^4^	*Wolbachia*	9e^−34^	none	—	Regulatory protein RepA
scaffold1344.1	1.9e^4^	*Citrobacter*	2e^−23^	none	—	Ig-like protein, Invasin
C2660673.1	2.3e^3^	*Lactobacillus*	1e^−53^	*Ixodes scapularis* (Ixodida)	6e^−22^	Permease
