# Supplementary material for: Draft genome of the most devastating insect pest of coffee worldwide: the coffee berry borer, Hypothenemus hampei
Source: Sci Rep. 2015 Jul 31;5:12525. doi: 10.1038/srep12525 (PMC4521149; doi:10.1038/srep12525)
Supplement: Supplementary Information [file srep12525-s1.pdf]

Supplementary Information

Draft genome of the most devastating insect pest of coffee worldwide: the coffee berry borer, *Hypothenemus hampei*

Fernando E. Vega, Stuart M. Brown, Hao Chen, Eric Shen, Mridul B. Nair, Javier A. Ceja-Navarro, Eoin L. Brodie, Francisco Infante, Patrick F. Dowd, and Arnab Pain

Table S1. Additional DNA and RNA assembly statistics.

**DNA**	
Mean size	1,876
Median size	127
Longest sequence	440,081
Shortest sequence	100
Singleton #	78,668
Average length of break (N) in scaffold	72
Scaffolds >1K	8,184 (9.4%)
Scaffolds >10K	3,205 (3.7%)
Scaffolds >100K	245 (0.28%)
Number of contigs	143068
Longest contig, bp	106114
Number of scaffolds > 1000 bp	8184
Number of scaffolds >N50	891
**RNA (polyA + RNA)**	
Scaffold number	54,068
Mean size	531
Median size	156
Longest sequence	12,501
Shortest sequence	100
Singleton #	78,668
Average length of break (N) in scaffold	7
Scaffolds >500	13,210 (24.4%)
Scaffolds >1K	8.742 (16.2%)

Table S2. Non-coding RNA predicted on the *H. hampei* draft genome by Infernal 1.1 using the Rfam database. Loci are reported by location on the draft genome scaffolds and contigs. E-values are shown for matches to Rfam models as reported by Infernal.

Name	Rfam Profil	Genome locus	start	end	strand	e-value	description
5S_rRNA	RF00001	scaffold5646	11516	11403	-	0.00097	5S ribosomal RNA
5S_rRNA	RF00001	scaffold6275	7509	7396	-	0.0026	5S ribosomal RNA
5S_rRNA	RF00001	C2512065	141	51	-	0.00019	5S ribosomal RNA
5S_rRNA	RF00001	C2663579	1095	1203	+	2.40E-17	5S ribosomal RNA
6S	RF00013	scaffold9366	34550	34357	-	2.00E-11	6S / SsrS RNA
Alfamo_CPB	RF00252	C2501025	181	113	-	0.0092	Alfalfa mosaic virus coat protein binding (CPB) I
AniS	RF02274	C2413626	7	70	+	0.0054	AniS
Archaea_SRP	RF01857	scaffold326	109728	109411	-	9.90E-13	Archaeal signal recognition particle RNA
SSU_rRNA_ar	RF01959	C2467619	145	1	-	2.20E-23	Archaeal small subunit ribosomal RNA
SSU_rRNA_ar	RF01959	C2573880	1	246	+	1.40E-46	Archaeal small subunit ribosomal RNA
SSU_rRNA_ar	RF01959	C2660673	49	1149	+	2.40E-210	Archaeal small subunit ribosomal RNA
Arthropod_7:	RF01052	scaffold167	44817	44568	-	3.80E-39	Arthropod 7SK RNA
HPnc0260	RF02194	scaffold7868	1961	2079	+	0.0022	Bacterial antisene RNA HPnc0260
Bacteria_larg	RF01854	scaffold10187	6734	6832	+	3.00E-10	Bacterial large signal recognition particle RNA
RNaseP_bact	RF00010	scaffold5463	10355	9933	-	6.60E-08	Bacterial RNase P class A
RNaseP_bact	RF00011	scaffold5463	10334	9944	-	2.10E-81	Bacterial RNase P class B
Bacteria_sma	RF00169	scaffold10187	6736	6831	+	2.00E-15	Bacterial small signal recognition particle RNA
SSU_rRNA_ba	RF00177	C2393994	1	101	+	1.50E-19	Bacterial small subunit ribosomal RNA
SSU_rRNA_ba	RF00177	C2396440	101	1	-	8.80E-15	Bacterial small subunit ribosomal RNA
SSU_rRNA_ba	RF00177	C2467619	145	1	-	9.00E-38	Bacterial small subunit ribosomal RNA
SSU_rRNA_ba	RF00177	C2573880	1	248	+	9.20E-81	Bacterial small subunit ribosomal RNA
SSU_rRNA_ba	RF00177	C2660673	44	1149	+	0	Bacterial small subunit ribosomal RNA
IRES_Bip	RF00223	scaffold4188	19736	19631	-	0.0065	bip internal ribosome entry site (IRES)
BsrG	RF01412	scaffold5346	2096	2332	+	5.70E-14	BsrG
BsrG	RF01412	C2622727	359	277	-	0.0016	BsrG
Cardiovirus_(	RF00453	C2451848	33	67	+	0.00096	Cardiovirus cis-acting replication element (CRE)
CC0734	RF01520	scaffold7168	1608	1551	-	0.0066	caulobacter sRNA CC0734
CC3510	RF01527	C2391032	1	92	+	0.001	caulobacter sRNA CC3510
Chlorobi-1	RF01696	C2707023	2360	2425	+	0.0051	Chlorobi-1 RNA
Telomerase-c	RF00025	C2468571	97	5	-	0.002	Ciliate telomerase RNA
class_I_RNA	RF01414	scaffold9300	16401	16455	+	0.0031	Class I RNA
CRISPR-DR8	RF01321	scaffold1764	13196	13230	+	0.002	CRISPR RNA direct repeat element
CRISPR-DR41	RF01350	scaffold4065	20838	20810	-	0.0076	CRISPR RNA direct repeat element
CRISPR-DR58	RF01371	scaffold9689	1095	1129	+	0.00037	CRISPR RNA direct repeat element
CRISPR-DR14	RF01327	C2391208	81	52	-	0.0048	CRISPR RNA direct repeat element

CRISPR-DR14	RF01327	C2395240	1	23	+	0.0047	CRISPR RNA direct repeat element
CRISPR-DR8	RF01321	C2395550	44	11	-	0.0034	CRISPR RNA direct repeat element
CRISPR-DR22	RF01335	C2397514	91	54	-	5.70E-05	CRISPR RNA direct repeat element
CRISPR-DR58	RF01371	C2457445	104	70	-	0.0096	CRISPR RNA direct repeat element
CRISPR-DR22	RF01335	C2460409	125	102	-	0.0091	CRISPR RNA direct repeat element
CRISPR-DR45	RF01354	C2481733	41	64	+	0.0052	CRISPR RNA direct repeat element
CRISPR-DR33	RF01343	C2482297	137	101	-	0.0037	CRISPR RNA direct repeat element
CRISPR-DR21	RF01334	C2553078	144	108	-	0.0089	CRISPR RNA direct repeat element
CRISPR-DR58	RF01371	C2554040	225	261	+	0.0077	CRISPR RNA direct repeat element
CRISPR-DR55	RF01368	C2643593	112	144	+	0.00078	CRISPR RNA direct repeat element
cspA	RF01766	scaffold8667	29042	29359	+	3.10E-13	cspA thermoregulator
CsrC	RF00084	scaffold9315	384	345	-	0.0013	CsrC RNA family
CsrC	RF00084	C2427378	70	35	-	0.00012	CsrC RNA family
CsrC	RF00084	C2544514	91	130	+	0.00073	CsrC RNA family
ctRNA_pT181	RF00242	scaffold5903	1840	1755	-	0.0066	ctRNA
DAOA-AS1_1	RF02090	scaffold4791	1211	1344	+	0.0064	DAOA antisense RNA 1 conserved region 1
rox2	RF01666	scaffold1476	181	231	+	2.60E-05	Drosophila rox2 ncRNA
rox2	RF01666	scaffold2905	26665	26725	+	0.001	Drosophila rox2 ncRNA
rox2	RF01666	scaffold5000	100224	100284	+	0.00015	Drosophila rox2 ncRNA
rox2	RF01666	scaffold5433	15044	15113	+	0.0031	Drosophila rox2 ncRNA
rox2	RF01666	C2433444	23	82	+	0.001	Drosophila rox2 ncRNA
rox2	RF01666	C2450520	111	27	-	0.0013	Drosophila rox2 ncRNA
rox2	RF01666	C2450564	9	72	+	0.006	Drosophila rox2 ncRNA
rox2	RF01666	C2490329	131	53	-	0.00036	Drosophila rox2 ncRNA
STnc410	RF02060	C2555450	174	274	+	0.0031	Enterobacterial sRNA STnc410
STnc430	RF02053	C2457083	1	71	+	0.007	Enterobacterial sRNA STnc430
STnc550	RF02081	C2416722	19	110	+	0.0088	Enterobacterial sRNA STnc550
Entero_OriR	RF00041	C2501727	28	154	+	0.004	Enteroviral 3' UTR element
Entero_OriR	RF00041	C2601184	151	42	-	0.0042	Enteroviral 3' UTR element
SSU_rRNA_eu	RF01960	C2390976	100	1	-	5.10E-20	Eukaryotic small subunit ribosomal RNA
SSU_rRNA_eu	RF01960	C2467619	145	1	-	2.60E-11	Eukaryotic small subunit ribosomal RNA
SSU_rRNA_eu	RF01960	C2573880	1	243	+	9.80E-40	Eukaryotic small subunit ribosomal RNA
SSU_rRNA_eu	RF01960	C2660673	49	1149	+	6.20E-111	Eukaryotic small subunit ribosomal RNA
Fungi_SRP	RF01502	scaffold326	109696	109415	-	0.0018	Fungal signal recognition particle RNA
Fungi_U3	RF01846	scaffold5609	7559	7429	-	0.002	Fungal small nucleolar RNA U3
rimP	RF01770	C2477775	98	18	-	0.0098	Gammaprotebacteria rimP leader

STnc400	RF02058	C2443488	113	2	-	1.60E-06	Gammaproteobacterial sRNA STnc400
GIR1	RF01807	C2484895	162	111	-	0.0048	GIR1 branching ribozyme
glmS	RF00234	scaffold8089	10696	10844	+	2.60E-28	glmS glucosamine-6-phosphate activated riboz
Intron_gpII	RF00029	scaffold4462	13438	13509	+	0.0005	Group II catalytic intron
Intron_gpII	RF00029	scaffold9403	3446	3349	-	3.20E-09	Group II catalytic intron
Intron_gpII	RF00029	scaffold10033	900	976	+	3.30E-05	Group II catalytic intron
Intron_gpII	RF00029	scaffold10950	14810	14909	+	4.10E-08	Group II catalytic intron
Intron_gpII	RF00029	C2398752	101	22	-	1.90E-08	Group II catalytic intron
group-II-D1D	RF01998	scaffold10187	16242	16170	-	8.10E-07	Group II catalytic intron D1-D4-1
group-II-D1D	RF02001	scaffold4462	1321	1150	-	1.30E-07	Group II catalytic intron D1-D4-3
group-II-D1D	RF02001	scaffold6204	16293	16460	+	3.10E-07	Group II catalytic intron D1-D4-3
group-II-D1D	RF02001	scaffold7161	91334	91495	+	5.10E-08	Group II catalytic intron D1-D4-3
group-II-D1D	RF02001	scaffold10187	16439	16268	-	5.60E-12	Group II catalytic intron D1-D4-3
group-II-D1D	RF02004	scaffold10158	384	175	-	8.20E-29	Group II catalytic intron D1-D4-5
group-II-D1D	RF02004	scaffold10950	14222	14421	+	1.90E-42	Group II catalytic intron D1-D4-5
Hammerheac	RF00163	C2411662	3	45	+	0.00062	Hammerhead ribozyme (type I)
Hammerheac	RF00008	scaffold4380	645	589	-	0.00012	Hammerhead ribozyme (type III)
Hammerheac	RF00008	scaffold4660	3944	3888	-	6.70E-06	Hammerhead ribozyme (type III)
Hammerheac	RF00008	scaffold4958	3141	3197	+	7.30E-06	Hammerhead ribozyme (type III)
Hammerheac	RF00008	scaffold7432	19110	19166	+	2.80E-05	Hammerhead ribozyme (type III)
Hammerheac	RF00008	C2621831	141	197	+	4.40E-07	Hammerhead ribozyme (type III)
Hammerheac	RF02275	C2525429	101	32	-	0.00071	Hammerhead ribozyme HH9
Histone3	RF00032	scaffold539	5233	5279	+	0.00021	Histone 3' UTR stem-loop
Histone3	RF00032	scaffold873	5548	5592	+	0.00095	Histone 3' UTR stem-loop
Histone3	RF00032	scaffold922	18813	18858	+	0.0073	Histone 3' UTR stem-loop
Histone3	RF00032	scaffold1092	62066	62111	+	0.00019	Histone 3' UTR stem-loop
Histone3	RF00032	scaffold3168	6301	6256	-	1.50E-05	Histone 3' UTR stem-loop
Histone3	RF00032	scaffold3600	13027	12983	-	0.00023	Histone 3' UTR stem-loop
Histone3	RF00032	scaffold3757	5564	5519	-	0.0015	Histone 3' UTR stem-loop
Histone3	RF00032	scaffold4166	72	27	-	0.00018	Histone 3' UTR stem-loop
Histone3	RF00032	scaffold4185	5235	5190	-	0.0096	Histone 3' UTR stem-loop
Histone3	RF00032	scaffold4399	1670	1714	+	0.0011	Histone 3' UTR stem-loop
Histone3	RF00032	scaffold4795	3345	3305	-	0.0022	Histone 3' UTR stem-loop
Histone3	RF00032	scaffold4995	2555	2600	+	0.0078	Histone 3' UTR stem-loop
Histone3	RF00032	scaffold5638	2078	2033	-	0.0014	Histone 3' UTR stem-loop
Histone3	RF00032	scaffold5829	76	31	-	9.20E-06	Histone 3' UTR stem-loop

Histone3	RF00032	scaffold5980	13454	13499	+	0.001	Histone 3' UTR stem-loop
Histone3	RF00032	scaffold6080	113	157	+	0.0032	Histone 3' UTR stem-loop
Histone3	RF00032	scaffold6099	843	888	+	0.00024	Histone 3' UTR stem-loop
Histone3	RF00032	scaffold6381	2649	2693	+	8.00E-05	Histone 3' UTR stem-loop
Histone3	RF00032	scaffold8916	777	822	+	0.0073	Histone 3' UTR stem-loop
Histone3	RF00032	scaffold9500	3067	3112	+	0.00016	Histone 3' UTR stem-loop
Histone3	RF00032	C2408580	19	64	+	0.0016	Histone 3' UTR stem-loop
Histone3	RF00032	C2409392	76	3	-	0.0059	Histone 3' UTR stem-loop
Histone3	RF00032	C2423598	2	42	+	4.50E-06	Histone 3' UTR stem-loop
Histone3	RF00032	C2427664	96	116	+	0.00058	Histone 3' UTR stem-loop
Histone3	RF00032	C2431940	46	2	-	5.90E-08	Histone 3' UTR stem-loop
Histone3	RF00032	C2433394	31	75	+	7.30E-07	Histone 3' UTR stem-loop
Histone3	RF00032	C2435204	100	120	+	0.0002	Histone 3' UTR stem-loop
Histone3	RF00032	C2437478	73	119	+	4.90E-06	Histone 3' UTR stem-loop
Histone3	RF00032	C2464191	4	50	+	0.0066	Histone 3' UTR stem-loop
Histone3	RF00032	C2499635	26	70	+	0.0016	Histone 3' UTR stem-loop
Histone3	RF00032	C2499675	26	70	+	0.0021	Histone 3' UTR stem-loop
Histone3	RF00032	C2502249	156	183	+	0.00016	Histone 3' UTR stem-loop
Histone3	RF00032	C2546308	241	267	+	0.00018	Histone 3' UTR stem-loop
Histone3	RF00032	C2602366	426	470	+	1.10E-06	Histone 3' UTR stem-loop
Histone3	RF00032	C2602366	106	62	-	8.60E-05	Histone 3' UTR stem-loop
Histone3	RF00032	C2633855	660	615	-	0.0076	Histone 3' UTR stem-loop
HIV_POL-1_S	RF01418	scaffold3563	8947	8845	-	0.0046	HIV pol-1 stem loop
HIV-1_SL3	RF01381	scaffold10302	2812	2790	-	0.0083	HIV-1 stem-loop 3 Psi packaging signal
HOXB13-AS1_	RF02133	scaffold5758	585	663	+	0.0043	HOXB13 antisense RNA 1 conserved region 2
IS1222_FSE	RF00383	scaffold9588	549	432	-	1.40E-26	Insertion sequence IS1222 ribosomal frameshif
IRE_I	RF00037	scaffold6049	1380	1340	-	0.0069	Iron response element I
IRE_I	RF00037	scaffold10935	1314	1274	-	0.0066	Iron response element I
IS009	RF02111	scaffold3980	1870	1798	-	0.0056	IS009
IS128	RF00125	C2538405	248	139	-	0.0014	IS128 RNA
isrG	RF01390	scaffold8082	7804	7859	+	0.0031	isrG Hfq binding RNA
isrJ	RF01393	C2506939	78	9	-	0.0033	isrJ Hfq binding RNA
isrK	RF01394	C2412058	108	57	-	0.0095	isrK Hfq binding RNA
lactis-plasmic	RF01742	C2554834	159	59	-	0.00067	lactis-plasmid RNA
LhrC	RF00616	C2397648	17	78	+	0.0044	Listeria Hfq binding LhrC
LhrC	RF00616	C2538405	219	100	-	0.0061	Listeria Hfq binding LhrC

rli51	RF01490	C2406316	1	105	+	0.0036	Listeria snRNA rli51
rli28	RF01492	scaffold5346	2072	2219	+	9.90E-05	Listeria sRNA rli28
rli28	RF01492	scaffold9525	13763	13669	-	0.005	Listeria sRNA rli28
rli28	RF01492	scaffold9807	134	1	-	4.50E-05	Listeria sRNA rli28
rli28	RF01492	C2474361	103	1	-	3.60E-06	Listeria sRNA rli28
rli38	RF01470	scaffold5346	29866	29733	-	0.00068	Listeria sRNA rli38
rli40	RF01472	C2648287	832	905	+	0.0008	Listeria sRNA rli40
rli52	RF01480	C2456695	118	78	-	0.0042	Listeria sRNA rli52
rli52	RF01480	C2565696	66	159	+	0.0027	Listeria sRNA rli52
rli61	RF01485	C2557992	222	128	-	0.00062	Listeria sRNA rli61
rliB	RF01471	C2499151	1	97	+	0.0055	Listeria sRNA rliB
rliD	RF01494	C2433444	22	81	+	1.70E-08	Listeria sRNA rliD
rliD	RF01494	C2433444	83	24	-	6.40E-08	Listeria sRNA rliD
rliE	RF01459	C2531031	40	98	+	0.0057	Listeria sRNA rliE
rliF	RF01476	scaffold8434	645	814	+	0.0083	Listeria sRNA rliF
Lysine	RF00168	scaffold8089	36919	37092	+	1.30E-27	Lysine riboswitch
Lysine	RF00168	scaffold10908	81401	81227	-	2.30E-22	Lysine riboswitch
mascRNA-me	RF01684	C2393834	7	77	+	2.50E-07	MALAT1-associated small cytoplasmic RNA/ME
Metazoa_SRI	RF00017	scaffold326	109712	109418	-	1.20E-71	Metazoan signal recognition particle RNA
Metazoa_SRI	RF00017	C2389038	79	1	-	3.20E-06	Metazoan signal recognition particle RNA
Metazoa_SRI	RF00017	C2390900	100	1	-	0.0043	Metazoan signal recognition particle RNA
Metazoa_SRI	RF00017	C2420622	63	1	-	3.10E-06	Metazoan signal recognition particle RNA
Metazoa_SRI	RF00017	C2471423	145	31	-	2.30E-09	Metazoan signal recognition particle RNA
Metazoa_SRI	RF00017	C2477917	155	43	-	0.0035	Metazoan signal recognition particle RNA
Metazoa_SRI	RF00017	C2509839	194	89	-	1.00E-08	Metazoan signal recognition particle RNA
Metazoa_SRI	RF00017	C2509839	69	1	-	3.70E-08	Metazoan signal recognition particle RNA
bantam	RF00727	scaffold2174	3898	3978	+	0.002	microRNA bantam
bantam	RF00727	scaffold3594	37430	37341	-	2.00E-16	microRNA bantam
bantam	RF00727	scaffold3934	611	699	+	0.00014	microRNA bantam
hvt-mir-H	RF01940	scaffold4374	36519	36435	-	0.00027	microRNA hvt-mir-H9
let-7	RF00027	scaffold465	23098	23018	-	1.50E-05	microRNA let-7 microRNA precursor
let-7	RF00027	scaffold836	84696	84745	+	0.0035	microRNA let-7 microRNA precursor
let-7	RF00027	scaffold3474	42370	42297	-	5.90E-10	microRNA let-7 microRNA precursor
let-7	RF00027	scaffold3474	42297	42370	+	0.0011	microRNA let-7 microRNA precursor
let-7	RF00027	scaffold10966	5188	5245	+	0.00016	microRNA let-7 microRNA precursor
let-7	RF00027	C2415674	107	18	-	0.0088	microRNA let-7 microRNA precursor

let-7	RF00027	C2511295	147	74	-	2.20E-06	microRNA let-7 microRNA precursor
lin-4	RF00052	scaffold3474	42150	42084	-	9.80E-08	microRNA lin-4 microRNA precursor
lin-4	RF00052	scaffold3474	42082	42149	+	0.00072	microRNA lin-4 microRNA precursor
lsy-6	RF00823	scaffold6661	3629	3559	-	0.002	microRNA lsy-6
lsy-6	RF00823	C2433444	19	87	+	2.50E-05	microRNA lsy-6
MIR1023	RF01043	scaffold8240	804	723	-	0.0017	microRNA MIR1023
MIR1027	RF00925	C2390636	99	4	-	1.00E-05	microRNA MIR1027
MIR1027	RF00925	C2390636	4	99	+	0.0056	microRNA MIR1027
MIR1027	RF00925	C2433444	103	1	-	1.00E-05	microRNA MIR1027
mir-11	RF00813	scaffold3315	36955	36890	-	9.50E-09	microRNA mir-11
mir-11	RF00813	scaffold4846	29422	29494	+	0.0037	microRNA mir-11
MIR1122	RF00906	scaffold91	33238	33330	+	0.00079	microRNA MIR1122
MIR1122	RF00906	scaffold91	33330	33238	-	0.0033	microRNA MIR1122
MIR1122	RF00906	scaffold684	71962	72040	+	4.90E-07	microRNA MIR1122
MIR1122	RF00906	scaffold684	72040	71962	-	2.10E-06	microRNA MIR1122
MIR1122	RF00906	scaffold1051	16657	16565	-	0.0018	microRNA MIR1122
MIR1122	RF00906	scaffold1051	16565	16657	+	0.0025	microRNA MIR1122
MIR1122	RF00906	scaffold1312	16507	16599	+	0.0071	microRNA MIR1122
MIR1122	RF00906	scaffold1316	20196	20287	+	0.0002	microRNA MIR1122
MIR1122	RF00906	scaffold1316	20287	20196	-	0.001	microRNA MIR1122
MIR1122	RF00906	scaffold1329	30063	30169	+	0.00057	microRNA MIR1122
MIR1122	RF00906	scaffold3251	1383	1232	-	4.00E-06	microRNA MIR1122
MIR1122	RF00906	scaffold3658	26	117	+	1.50E-06	microRNA MIR1122
MIR1122	RF00906	scaffold3658	117	26	-	2.80E-06	microRNA MIR1122
MIR1122	RF00906	scaffold3893	14555	14463	-	6.20E-05	microRNA MIR1122
MIR1122	RF00906	scaffold3893	14463	14555	+	0.0072	microRNA MIR1122
MIR1122	RF00906	scaffold5229	35385	35478	+	0.006	microRNA MIR1122
MIR1122	RF00906	scaffold6920	11163	11051	-	0.0042	microRNA MIR1122
MIR1122	RF00906	scaffold6955	15909	15817	-	3.30E-06	microRNA MIR1122
MIR1122	RF00906	scaffold6955	15817	15909	+	8.80E-06	microRNA MIR1122
MIR1122	RF00906	scaffold8385	3412	3537	+	1.90E-05	microRNA MIR1122
MIR1122	RF00906	scaffold8385	3537	3412	-	7.00E-05	microRNA MIR1122
MIR1122	RF00906	scaffold9033	433	542	+	8.40E-05	microRNA MIR1122
MIR1122	RF00906	C2410734	105	3	-	0.0071	microRNA MIR1122
MIR1122	RF00906	C2467781	124	32	-	5.10E-06	microRNA MIR1122
MIR1122	RF00906	C2467781	32	124	+	8.90E-06	microRNA MIR1122

MIR1122	RF00906	C2538405	209	145	-	0.0026	microRNA MIR1122
mir-12	RF00751	scaffold42	38403	38335	-	7.60E-06	microRNA mir-12
mir-12	RF00751	C2390636	5	98	+	0.0085	microRNA mir-12
mir-1249	RF01918	scaffold2124	10291	10197	-	0.00027	microRNA mir-1249
mir-126	RF00701	scaffold2935	32917	32845	-	0.0022	microRNA mir-126
mir-132	RF00662	scaffold11034	5312	5236	-	0.00053	microRNA mir-132
mir-137	RF00694	scaffold3261	14532	14434	-	1.10E-19	microRNA mir-137
mir-14	RF00752	scaffold5217	7940	7877	-	3.60E-06	microRNA mir-14
mir-14	RF00752	scaffold5419	10910	10855	-	0.0065	microRNA mir-14
mir-14	RF00752	C2449896	78	124	+	0.0026	microRNA mir-14
mir-14	RF00752	C2528141	39	94	+	0.0014	microRNA mir-14
mir-140	RF00678	C2428676	117	43	-	0.00013	microRNA mir-140
mir-142	RF01896	C2398502	18	101	+	1.60E-06	microRNA mir-142
MIR1444	RF00956	scaffold4672	3344	3262	-	0.001	microRNA MIR1444
MIR1444	RF00956	C2439268	2	118	+	4.30E-05	microRNA MIR1444
MIR1444	RF00956	C2443488	107	44	-	0.003	microRNA MIR1444
MIR1446	RF00954	scaffold10427	830	926	+	0.0027	microRNA MIR1446
MIR1446	RF00954	C2487903	84	165	+	0.00069	microRNA MIR1446
mir-145	RF00675	C2464747	62	141	+	0.0065	microRNA mir-145
mir-154	RF00641	scaffold2891	6807	6864	+	0.0009	microRNA mir-154
mir-155	RF00731	C2398502	1	63	+	0.00091	microRNA mir-155
MIR162_2	RF00742	scaffold100	14535	14622	+	1.60E-05	microRNA MIR162_2
MIR162_2	RF00742	scaffold3507	1467	1378	-	0.00036	microRNA MIR162_2
MIR162_2	RF00742	C2450520	113	26	-	0.00083	microRNA MIR162_2
MIR162_2	RF00742	C2454758	34	100	+	0.00044	microRNA MIR162_2
MIR162_2	RF00742	C2454758	101	35	-	0.00044	microRNA MIR162_2
MIR162_2	RF00742	C2533709	145	197	+	7.20E-05	microRNA MIR162_2
MIR164	RF00647	scaffold5356	10709	10609	-	6.10E-06	microRNA MIR164
MIR167_1	RF00640	scaffold3086	71948	71841	-	0.0019	microRNA MIR167_1
mir-184	RF00657	scaffold1467	123647	123726	+	6.30E-11	microRNA mir-184
mir-184	RF00657	scaffold1467	124198	124276	+	0.0042	microRNA mir-184
mir-185	RF00771	C2449896	77	125	+	0.0023	microRNA mir-185
mir-185	RF00771	C2528141	28	106	+	4.90E-05	microRNA mir-185
mir-186	RF00697	scaffold1968	8347	8256	-	0.0023	microRNA mir-186
mir-186	RF00697	scaffold7467	477	603	+	0.009	microRNA mir-186
mir-186	RF00697	C2433444	88	18	-	0.0048	microRNA mir-186

mir-186	RF00697	C2451162	112	22	-	1.60E-05	microRNA mir-186
mir-186	RF00697	C2497697	155	24	-	0.0005	microRNA mir-186
mir-187	RF00674	C2505267	1	96	+	0.00039	microRNA mir-187
mir-190	RF00672	scaffold1590	20198	20113	-	8.90E-15	microRNA mir-190
mir-190	RF00672	scaffold1746	8830	8760	-	0.0045	microRNA mir-190
mir-190	RF00672	scaffold6915	557	648	+	2.60E-06	microRNA mir-190
mir-190	RF00672	scaffold6915	648	557	-	0.00011	microRNA mir-190
mir-1912	RF02017	scaffold549	130	204	+	6.70E-05	microRNA mir-1912
mir-1912	RF02017	scaffold549	201	127	-	6.80E-05	microRNA mir-1912
mir-1912	RF02017	C2398502	62	2	-	1.40E-06	microRNA mir-1912
mir-1912	RF02017	C2467781	42	117	+	9.50E-05	microRNA mir-1912
mir-1912	RF02017	C2501727	43	115	+	0.00035	microRNA mir-1912
mir-1912	RF02017	C2501727	110	38	-	0.0095	microRNA mir-1912
mir-197	RF00707	C2449896	78	124	+	0.00043	microRNA mir-197
mir-202	RF00705	C2405622	85	3	-	9.50E-09	microRNA mir-202
mir-202	RF00705	C2405622	6	88	+	8.40E-08	microRNA mir-202
mir-2024	RF01900	C2398502	1	77	+	0.00089	microRNA mir-2024
mir-203	RF00696	scaffold5827	6731	6799	+	3.40E-05	microRNA mir-203
mir-203	RF00696	scaffold5827	6803	6735	-	0.0021	microRNA mir-203
mir-208	RF00749	C2588046	289	367	+	0.00016	microRNA mir-208
mir-210	RF00679	scaffold5229	10428	10333	-	9.90E-16	microRNA mir-210
mir-210	RF00679	scaffold5229	10343	10438	+	3.20E-06	microRNA mir-210
mir-217	RF00673	C2525421	61	154	+	0.0065	microRNA mir-217
mir-2238	RF01915	C2554834	163	69	-	0.0005	microRNA mir-2238
mir-231	RF00852	scaffold3843	4656	4729	+	0.00013	microRNA mir-231
mir-231	RF00852	C2405622	9	82	+	5.10E-06	microRNA mir-231
mir-231	RF00852	C2405622	82	9	-	1.20E-05	microRNA mir-231
mir-232	RF00856	C2459249	15	119	+	0.0055	microRNA mir-232
mir-233	RF00857	scaffold5297	9634	9727	+	0.0097	microRNA mir-233
mir-233	RF00857	C2405622	1	89	+	6.90E-05	microRNA mir-233
mir-240	RF00804	scaffold4588	3556	3668	+	4.90E-06	microRNA mir-240
mir-241	RF00809	scaffold1316	17783	17697	-	0.00024	microRNA mir-241
mir-241	RF00809	C2525421	73	156	+	0.0039	microRNA mir-241
mir-241	RF00809	C2528141	24	112	+	0.00063	microRNA mir-241
mir-242	RF00898	scaffold116	24228	24288	+	0.002	microRNA mir-242
mir-242	RF00898	C2533709	202	141	-	0.0021	microRNA mir-242

mir-244	RF00815	C2433444	91	13	-	0.0017	microRNA mir-244
mir-244	RF00815	C2478273	134	66	-	0.0057	microRNA mir-244
mir-246	RF00819	scaffold2933	7184	7277	+	0.0012	microRNA mir-246
mir-248	RF00820	scaffold8293	1861	1756	-	0.0075	microRNA mir-248
mir-249	RF00821	scaffold4366	7405	7304	-	0.0033	microRNA mir-249
mir-249	RF00821	C2433444	109	10	-	1.10E-06	microRNA mir-249
mir-249	RF00821	C2433444	1	95	+	3.50E-06	microRNA mir-249
mir-25	RF02020	scaffold1707	48012	47931	-	0.0019	microRNA mir-25
mir-251	RF00837	scaffold3251	1265	1352	+	0.00061	microRNA mir-251
mir-252	RF00838	scaffold3359	13899	14000	+	1.00E-12	microRNA mir-252
mir-252	RF00838	scaffold3359	13997	13905	-	0.00011	microRNA mir-252
mir-255	RF00900	C2443488	19	119	+	0.007	microRNA mir-255
mir-259	RF00850	scaffold537	25611	25518	-	0.00015	microRNA mir-259
mir-263	RF00706	scaffold1502	25676	25762	+	4.40E-13	microRNA mir-263
mir-263	RF00706	scaffold2696	66827	66913	+	5.00E-12	microRNA mir-263
mir-263	RF00706	scaffold2696	66912	66828	-	0.0034	microRNA mir-263
mir-268	RF00834	scaffold6259	887	803	-	0.0068	microRNA mir-268
mir-268	RF00834	scaffold10966	5259	5176	-	6.80E-05	microRNA mir-268
mir-268	RF00834	scaffold10966	5173	5260	+	0.00023	microRNA mir-268
mir-268	RF00834	C2456695	57	136	+	0.0085	microRNA mir-268
mir-275	RF00757	scaffold5674	5016	4926	-	9.50E-08	microRNA mir-275
mir-276	RF00637	scaffold1306	1450	1392	-	0.0026	microRNA mir-276
mir-276	RF00637	scaffold5195	2155	2064	-	0.0016	microRNA mir-276
mir-276	RF00637	scaffold9049	11942	11854	-	6.60E-20	microRNA mir-276
mir-276	RF00637	scaffold9049	11857	11947	+	1.70E-08	microRNA mir-276
mir-277	RF00730	scaffold2173	35487	35583	+	4.80E-12	microRNA mir-277
mir-279	RF00754	scaffold940	117256	117156	-	0.00013	microRNA mir-279
mir-279	RF00754	scaffold4925	21947	21857	-	3.80E-13	microRNA mir-279
mir-279	RF00754	scaffold4925	21801	21724	-	0.0011	microRNA mir-279
mir-279	RF00754	scaffold4925	21855	21945	+	0.0031	microRNA mir-279
mir-28	RF00655	scaffold985	13971	14055	+	0.00073	microRNA mir-28
mir-280	RF00801	C2405622	78	12	-	7.60E-05	microRNA mir-280
mir-280	RF00801	C2494567	15	100	+	0.0064	microRNA mir-280
mir-282	RF00724	scaffold987	50466	50377	-	0.0055	microRNA mir-282
mir-282	RF00724	scaffold3379	15833	15742	-	1.10E-10	microRNA mir-282
mir-282	RF00724	scaffold3379	15742	15833	+	4.60E-06	microRNA mir-282

mir-283	RF00747	scaffold42	39344	39265	-	6.60E-08	microRNA mir-283
mir-283	RF00747	C2427894	112	15	-	0.0044	microRNA mir-283
mir-284	RF01901	C2456695	121	73	-	0.0068	microRNA mir-284
mir-286	RF00789	scaffold2158	985	885	-	2.20E-06	microRNA mir-286
mir-286	RF00789	scaffold2158	889	987	+	1.50E-05	microRNA mir-286
mir-286	RF00789	scaffold9771	82	2	-	2.40E-06	microRNA mir-286
mir-286	RF00789	scaffold9771	5	85	+	4.20E-06	microRNA mir-286
mir-286	RF00789	C2498069	12	92	+	0.0069	microRNA mir-286
mir-287	RF00788	scaffold2098	67638	67550	-	0.0016	microRNA mir-287
mir-290	RF00665	scaffold8334	2811	2878	+	0.00016	microRNA mir-290
mir-290	RF00665	scaffold8334	2879	2810	-	0.0012	microRNA mir-290
mir-290	RF00665	C2441632	22	105	+	2.90E-05	microRNA mir-290
mir-290	RF00665	C2682855	1497	1544	+	0.0016	microRNA mir-290
mir-299	RF00756	scaffold3228	3548	3609	+	0.0001	microRNA mir-299
mir-299	RF00756	scaffold3228	3609	3548	-	0.00015	microRNA mir-299
mir-3	RF00716	scaffold1725	3845	3913	+	0.00015	microRNA mir-3
mir-3	RF00716	scaffold5735	100	38	-	3.00E-05	microRNA mir-3
mir-3	RF00716	C2449288	117	15	-	0.00036	microRNA mir-3
mir-305	RF00732	scaffold5674	4334	4249	-	1.40E-16	microRNA mir-305
mir-305	RF00732	scaffold5674	4249	4334	+	3.40E-06	microRNA mir-305
mir-308	RF00743	scaffold158	17272	17339	+	4.50E-05	microRNA mir-308
mir-308	RF00743	scaffold9437	48	106	+	0.0041	microRNA mir-308
mir-308	RF00743	C2539605	105	146	+	8.90E-07	microRNA mir-308
mir-308	RF00743	C2539605	146	105	-	8.90E-07	microRNA mir-308
mir-31	RF00661	scaffold4948	3193	3114	-	2.30E-11	microRNA mir-31
mir-315	RF00717	scaffold6343	44244	44324	+	1.60E-09	microRNA mir-315
mir-315	RF00717	scaffold6343	44324	44244	-	0.0016	microRNA mir-315
mir-315	RF00717	scaffold6667	2679	2748	+	1.30E-05	microRNA mir-315
mir-315	RF00717	scaffold6667	2748	2679	-	1.50E-05	microRNA mir-315
mir-316	RF00814	scaffold4243	36792	36702	-	0.00079	microRNA mir-316
mir-317	RF00720	scaffold2173	34218	34304	+	3.00E-11	microRNA mir-317
mir-318	RF00818	C2458753	17	61	+	0.0064	microRNA mir-318
mir-326	RF00719	scaffold6995	47	137	+	3.60E-06	microRNA mir-326
mir-328	RF00772	C2449896	125	66	-	0.0016	microRNA mir-328
mir-328	RF00772	C2449896	77	131	+	0.0031	microRNA mir-328
mir-33	RF00667	scaffold3851	34788	34853	+	5.00E-10	microRNA mir-33

mir-33	RF00667	C2459249	107	28	-	2.90E-05	microRNA mir-33
mir-331	RF00769	scaffold974	29925	29984	+	0.0036	microRNA mir-331
mir-331	RF00769	scaffold1009	6402	6321	-	0.0044	microRNA mir-331
mir-335	RF00766	scaffold2032	4928	5018	+	4.80E-06	microRNA mir-335
mir-335	RF00766	C2528141	21	121	+	1.50E-05	microRNA mir-335
mir-340	RF00761	scaffold3266	1438	1299	-	0.009	microRNA mir-340
mir-340	RF00761	scaffold4348	7428	7516	+	0.00054	microRNA mir-340
mir-340	RF00761	scaffold10966	5258	5176	-	8.80E-09	microRNA mir-340
mir-345	RF01044	C2393834	1	73	+	0.002	microRNA mir-345
mir-353	RF00800	C2592280	180	250	+	0.0038	microRNA mir-353
mir-355	RF00797	scaffold1987	18683	18802	+	0.00074	microRNA mir-355
mir-355	RF00797	scaffold1987	18803	18690	-	0.0018	microRNA mir-355
mir-357	RF00791	C2405622	4	90	+	3.40E-08	microRNA mir-357
mir-357	RF00791	C2405622	87	4	-	6.80E-08	microRNA mir-357
mir-374	RF00840	C2470675	121	26	-	0.00096	microRNA mir-374
mir-384	RF00841	scaffold3102	5713	5789	+	0.0029	microRNA mir-384
mir-384	RF00841	scaffold8281	1554	1471	-	0.00027	microRNA mir-384
MIR390	RF00689	C2436062	7	121	+	0.006	microRNA MIR390
MIR390	RF00689	C2450520	7	131	+	0.0018	microRNA MIR390
mir-392	RF00904	scaffold2432	9549	9482	-	0.00081	microRNA mir-392
mir-392	RF00904	scaffold8273	611	545	-	2.50E-06	microRNA mir-392
MIR394	RF00688	scaffold234	34239	34346	+	0.0022	microRNA MIR394
MIR394	RF00688	scaffold234	34342	34243	-	0.0067	microRNA MIR394
MIR396	RF00648	scaffold745	10080	9921	-	1.10E-07	microRNA MIR396
MIR396	RF00648	scaffold2837	28067	27911	-	0.002	microRNA MIR396
MIR396	RF00648	scaffold10276	1569	1349	-	0.0025	microRNA MIR396
MIR396	RF00648	scaffold10276	1350	1570	+	0.0032	microRNA MIR396
MIR396	RF00648	C2405622	81	9	-	0.0065	microRNA MIR396
MIR396	RF00648	C2442252	13	104	+	0.0025	microRNA MIR396
MIR396	RF00648	C2451162	122	10	-	6.80E-05	microRNA MIR396
MIR396	RF00648	C2570198	165	228	+	0.002	microRNA MIR396
MIR397	RF00704	scaffold6766	31432	31515	+	0.00016	microRNA MIR397
MIR397	RF00704	C2459249	119	18	-	0.0019	microRNA MIR397
MIR397	RF00704	C2556840	195	77	-	0.0011	microRNA MIR397
MIR398	RF00695	C2492555	34	137	+	0.0027	microRNA MIR398
MIR398	RF00695	C2492555	137	34	-	0.003	microRNA MIR398

MIR398	RF00695	C2539605	99	152	+	4.90E-05	microRNA MIR398
MIR398	RF00695	C2539605	152	99	-	4.90E-05	microRNA MIR398
MIR403	RF00842	scaffold3958	103463	103539	+	0.0012	microRNA MIR403
MIR403	RF00842	C2448856	6	127	+	3.40E-05	microRNA MIR403
MIR405	RF00768	scaffold4260	1875	1996	+	0.0079	microRNA MIR405
MIR405	RF00768	C2538405	235	125	-	5.70E-05	microRNA MIR405
MIR405	RF00768	C2588046	383	283	-	0.0002	microRNA MIR405
MIR408	RF00690	scaffold466	44488	44329	-	0.00055	microRNA MIR408
mir-42	RF00794	scaffold1234	5125	5226	+	6.30E-07	microRNA mir-42
mir-42	RF00794	scaffold5448	23150	23233	+	0.00093	microRNA mir-42
mir-42	RF00794	C2450564	89	1	-	0.00032	microRNA mir-42
mir-423	RF00870	C2528141	29	104	+	5.40E-05	microRNA mir-423
mir-43	RF00795	C2539605	103	145	+	0.0015	microRNA mir-43
mir-43	RF00795	C2539605	148	106	-	0.0015	microRNA mir-43
mir-44	RF00710	C2451162	22	114	+	0.00021	microRNA mir-44
MIR444	RF00920	scaffold7492	3171	3030	-	1.00E-05	microRNA MIR444
MIR444	RF00920	C2415676	109	1	-	2.10E-08	microRNA MIR444
MIR444	RF00920	C2415676	2	109	+	2.10E-07	microRNA MIR444
MIR444	RF00920	C2439268	122	1	-	6.40E-05	microRNA MIR444
MIR444	RF00920	C2442528	125	2	-	0.00094	microRNA MIR444
MIR444	RF00920	C2450520	114	24	-	0.0019	microRNA MIR444
MIR444	RF00920	C2487903	65	165	+	0.001	microRNA MIR444
mir-449	RF00711	scaffold2173	35850	35933	+	6.20E-05	microRNA mir-449
mir-450	RF00708	C2494871	158	81	-	0.0044	microRNA mir-450
mir-451	RF00722	scaffold8563	5790	5720	-	0.00047	microRNA mir-451
mir-451	RF00722	scaffold8563	5726	5784	+	0.00053	microRNA mir-451
mir-451	RF00722	C2467291	127	56	-	0.00016	microRNA mir-451
mir-454	RF00746	C2398502	101	11	-	0.0092	microRNA mir-454
mir-458	RF00750	scaffold2032	4934	5007	+	5.20E-07	microRNA mir-458
mir-458	RF00750	C2398502	46	96	+	0.00086	microRNA mir-458
mir-458	RF00750	C2398502	96	46	-	0.00093	microRNA mir-458
mir-463	RF00934	C2400106	74	6	-	8.10E-05	microRNA mir-463
mir-463	RF00934	C2405622	17	74	+	9.80E-05	microRNA mir-463
mir-463	RF00934	C2454758	30	110	+	0.0035	microRNA mir-463
mir-463	RF00934	C2454758	105	25	-	0.0035	microRNA mir-463
mir-471	RF00932	C2456109	76	16	-	0.0063	microRNA mir-471

MIR475	RF00721	scaffold7148	3022	2943	-	0.0042	microRNA MIR475
MIR475	RF00721	C2492555	145	26	-	0.00021	microRNA MIR475
MIR476	RF00739	scaffold794	37503	37589	+	4.20E-07	microRNA MIR476
MIR476	RF00739	scaffold7453	1356	1311	-	0.00012	microRNA MIR476
MIR476	RF00739	C2428002	105	7	-	0.0094	microRNA MIR476
MIR476	RF00739	C2451162	108	17	-	0.0024	microRNA MIR476
MIR477	RF00780	scaffold414	356	282	-	0.0017	microRNA MIR477
MIR477	RF00780	scaffold516	263	358	+	0.0029	microRNA MIR477
MIR477	RF00780	scaffold1347	5420	5349	-	0.009	microRNA MIR477
MIR477	RF00780	C2433444	4	100	+	5.90E-10	microRNA MIR477
MIR477	RF00780	C2459249	13	121	+	0.00027	microRNA MIR477
MIR477	RF00780	C2464803	138	36	-	0.0051	microRNA MIR477
MIR478	RF00652	C2508183	183	121	-	0.0033	microRNA MIR478
mir-48	RF00796	scaffold714	41491	41583	+	3.50E-06	microRNA mir-48
mir-48	RF00796	scaffold2219	10024	10126	+	0.0003	microRNA mir-48
mir-48	RF00796	scaffold10966	5174	5269	+	6.90E-06	microRNA mir-48
mir-498	RF00958	scaffold21	234772	234652	-	7.00E-05	microRNA mir-498
mir-499	RF00745	C2525421	72	157	+	0.0074	microRNA mir-499
mir-5	RF00854	scaffold4846	29426	29492	+	0.0016	microRNA mir-5
mir-5	RF00854	scaffold4846	29492	29422	-	0.0023	microRNA mir-5
mir-5	RF00854	scaffold6995	62	124	+	0.00032	microRNA mir-5
mir-5	RF00854	C2398502	3	63	+	0.0074	microRNA mir-5
mir-50	RF00824	scaffold8168	93	1	-	0.0045	microRNA mir-50
mir-506	RF01910	C2405622	81	10	-	4.00E-07	microRNA mir-506
MIR529	RF00908	scaffold739	44711	44579	-	0.0063	microRNA MIR529
MIR529	RF00908	scaffold10966	5262	5170	-	5.20E-07	microRNA MIR529
MIR529	RF00908	C2415674	18	107	+	9.70E-05	microRNA MIR529
MIR529	RF00908	C2512027	16	158	+	8.80E-05	microRNA MIR529
MIR529	RF00908	C2512027	156	16	-	0.00014	microRNA MIR529
MIR530	RF01005	scaffold221	5576	5419	-	1.40E-05	microRNA MIR530
MIR530	RF01005	scaffold402	139735	139583	-	0.0066	microRNA MIR530
MIR530	RF01005	scaffold745	9925	10077	+	0.0011	microRNA MIR530
MIR530	RF01005	scaffold2240	11537	11694	+	0.008	microRNA MIR530
MIR530	RF01005	scaffold2725	12827	12989	+	0.0021	microRNA MIR530
MIR530	RF01005	scaffold4692	2457	2595	+	0.001	microRNA MIR530
MIR530	RF01005	scaffold4836	2488	2658	+	0.00011	microRNA MIR530

MIR530	RF01005	scaffold6180	81718	81854	+	0.0025	microRNA MIR530
MIR530	RF01005	C2405622	85	6	-	0.00042	microRNA MIR530
MIR530	RF01005	C2497697	165	13	-	2.60E-05	microRNA MIR530
MIR530	RF01005	C2565696	108	171	+	0.00064	microRNA MIR530
mir-548	RF01061	scaffold3340	41305	41387	+	0.00017	microRNA mir-548
mir-548	RF01061	C2399662	1	60	+	0.00062	microRNA mir-548
mir-548	RF01061	C2456695	76	120	+	0.00017	microRNA mir-548
mir-548	RF01061	C2458969	1	73	+	5.60E-05	microRNA mir-548
mir-548	RF01061	C2458969	73	1	-	0.00026	microRNA mir-548
mir-548	RF01061	C2467781	120	36	-	4.20E-07	microRNA mir-548
mir-549	RF00965	scaffold10595	2545	2479	-	0.0048	microRNA mir-549
mir-549	RF00965	C2565696	180	102	-	0.00018	microRNA mir-549
mir-55	RF00826	C2528141	19	108	+	6.80E-05	microRNA mir-55
mir-551	RF00892	scaffold797	2731	2816	+	0.0062	microRNA mir-551
mir-552	RF00990	scaffold7210	1248	1164	-	6.20E-05	microRNA mir-552
mir-552	RF00990	C2554962	170	75	-	1.40E-07	microRNA mir-552
mir-556	RF00969	scaffold1968	8342	8252	-	0.00014	microRNA mir-556
mir-556	RF00969	scaffold2010	1898	1807	-	2.80E-05	microRNA mir-556
mir-556	RF00969	scaffold2010	1820	1898	+	0.0023	microRNA mir-556
mir-556	RF00969	scaffold6259	874	816	-	7.10E-05	microRNA mir-556
mir-556	RF00969	scaffold6811	1036	946	-	0.0024	microRNA mir-556
mir-556	RF00969	scaffold6995	55	136	+	2.90E-05	microRNA mir-556
mir-556	RF00969	scaffold10132	8664	8574	-	0.0023	microRNA mir-556
mir-556	RF00969	C2515821	55	127	+	0.00096	microRNA mir-556
mir-562	RF00998	C2393566	1	65	+	0.0029	microRNA mir-562
mir-563	RF01003	scaffold9666	956	1020	+	0.0042	microRNA mir-563
mir-569	RF01018	C2443488	32	119	+	0.00045	microRNA mir-569
mir-577	RF01013	C2648287	861	924	+	0.0096	microRNA mir-577
mir-578	RF00971	scaffold10966	5266	5172	-	3.30E-12	microRNA mir-578
mir-578	RF00971	scaffold10966	5166	5260	+	1.80E-10	microRNA mir-578
mir-582	RF00927	C2498069	9	99	+	0.00098	microRNA mir-582
mir-583	RF00976	C2525777	187	113	-	0.00026	microRNA mir-583
mir-590	RF00928	scaffold2292	6559	6651	+	3.00E-06	microRNA mir-590
mir-592	RF00877	scaffold290	6553	6641	+	0.00015	microRNA mir-592
mir-598	RF01059	scaffold1204	40500	40575	+	0.0031	microRNA mir-598
mir-598	RF01059	scaffold1234	5228	5130	-	4.80E-10	microRNA mir-598

mir-598	RF01059	scaffold1234	5124	5222	+	2.70E-07	microRNA mir-598
mir-598	RF01059	scaffold1316	17699	17792	+	6.30E-05	microRNA mir-598
mir-598	RF01059	scaffold1316	17789	17699	-	0.0062	microRNA mir-598
mir-598	RF01059	scaffold4188	21633	21719	+	0.0021	microRNA mir-598
mir-598	RF01059	scaffold4190	48807	48691	-	9.90E-06	microRNA mir-598
mir-598	RF01059	scaffold4190	48697	48789	+	3.70E-05	microRNA mir-598
mir-598	RF01059	scaffold5239	18732	18816	+	0.00017	microRNA mir-598
mir-598	RF01059	scaffold5239	18819	18741	-	0.00075	microRNA mir-598
mir-598	RF01059	scaffold6407	18435	18487	+	0.0037	microRNA mir-598
mir-598	RF01059	scaffold9783	1134	1068	-	0.0011	microRNA mir-598
mir-598	RF01059	scaffold10966	5170	5257	+	1.80E-09	microRNA mir-598
mir-598	RF01059	C2454758	3	126	+	5.40E-08	microRNA mir-598
mir-598	RF01059	C2454758	132	9	-	5.40E-08	microRNA mir-598
mir-598	RF01059	C2458969	71	2	-	0.0017	microRNA mir-598
mir-598	RF01059	C2525421	154	79	-	1.30E-05	microRNA mir-598
mir-598	RF01059	C2525421	70	151	+	1.40E-05	microRNA mir-598
mir-605	RF01011	C2449252	28	74	+	0.0074	microRNA mir-605
mir-605	RF01011	C2490863	45	125	+	0.009	microRNA mir-605
mir-616	RF00995	C2433444	4	100	+	5.20E-06	microRNA mir-616
mir-628	RF01012	C2556338	227	136	-	0.0015	microRNA mir-628
mir-649	RF01029	C2431340	8	99	+	0.0023	microRNA mir-649
mir-649	RF01029	C2524459	63	156	+	0.00036	microRNA mir-649
mir-651	RF00972	scaffold1316	20280	20193	-	0.0064	microRNA mir-651
mir-651	RF00972	scaffold2287	2957	2899	-	0.00019	microRNA mir-651
mir-651	RF00972	C2405622	6	94	+	4.10E-09	microRNA mir-651
mir-651	RF00972	C2451162	98	26	-	0.0014	microRNA mir-651
mir-651	RF00972	C2451162	35	107	+	0.0044	microRNA mir-651
mir-653	RF00937	scaffold10050	3381	3477	+	0.0033	microRNA mir-653
mir-653	RF00937	C2433444	6	110	+	5.00E-11	microRNA mir-653
mir-653	RF00937	C2433444	95	10	-	1.20E-05	microRNA mir-653
mir-654	RF01922	C2505217	88	178	+	0.0049	microRNA mir-654
mir-665	RF00921	C2505267	15	95	+	0.0011	microRNA mir-665
mir-67	RF00844	scaffold2373	23286	23226	-	7.20E-13	microRNA mir-67
mir-67	RF00844	scaffold2373	23227	23287	+	0.00063	microRNA mir-67
mir-672	RF00911	C2405622	84	4	-	2.20E-05	microRNA mir-672
mir-672	RF00911	C2467781	127	32	-	0.0043	microRNA mir-672

mir-70	RF00833	C2545116	147	262	+	0.0074	microRNA mir-70
mir-71	RF00832	scaffold3143	14741	14796	+	2.30E-10	microRNA mir-71
mir-71	RF00832	scaffold3143	14797	14742	-	3.80E-05	microRNA mir-71
mir-764	RF01920	scaffold9771	99	1	-	0.0045	microRNA mir-764
mir-785	RF02244	scaffold5203	96	26	-	0.0084	microRNA mir-785
mir-785	RF02244	C2433444	9	96	+	7.20E-07	microRNA mir-785
mir-785	RF02244	C2451162	26	107	+	0.00061	microRNA mir-785
mir-785	RF02244	C2454758	21	114	+	1.50E-09	microRNA mir-785
mir-785	RF02244	C2454758	114	21	-	1.50E-09	microRNA mir-785
mir-785	RF02244	C2459249	27	108	+	0.006	microRNA mir-785
mir-785	RF02244	C2537313	211	115	-	0.0041	microRNA mir-785
mir-786	RF00895	C2451162	26	107	+	0.00047	microRNA mir-786
mir-787	RF00896	C2398502	101	32	-	0.00077	microRNA mir-787
mir-788	RF02245	C2400106	5	80	+	3.00E-05	microRNA mir-788
mir-789	RF00905	C2525421	142	85	-	1.00E-05	microRNA mir-789
MIR807	RF00886	scaffold5297	2782	2926	+	0.0011	microRNA MIR807
MIR807	RF00886	C2390888	96	15	-	0.0036	microRNA MIR807
mir-81	RF00728	scaffold3594	37436	37342	-	2.30E-08	microRNA mir-81
mir-81	RF00728	scaffold3594	37338	37434	+	0.0042	microRNA mir-81
MIR811	RF00882	C2450520	114	24	-	0.00057	microRNA MIR811
MIR811	RF00882	C2501727	29	124	+	0.0015	microRNA MIR811
MIR815	RF00884	scaffold729	5670	5746	+	0.0033	microRNA MIR815
MIR815	RF00884	C2400106	7	78	+	0.00041	microRNA MIR815
MIR821	RF00885	scaffold151	9264	9169	-	2.10E-05	microRNA MIR821
MIR821	RF00885	scaffold892	11424	11320	-	2.80E-06	microRNA MIR821
MIR821	RF00885	scaffold977	1488	1154	-	1.20E-15	microRNA MIR821
MIR821	RF00885	scaffold1320	3714	3815	+	6.90E-06	microRNA MIR821
MIR821	RF00885	scaffold1406	33304	33646	+	1.60E-09	microRNA MIR821
MIR821	RF00885	scaffold1741	9812	10001	+	1.00E-07	microRNA MIR821
MIR821	RF00885	scaffold2055	12953	13031	+	0.0058	microRNA MIR821
MIR821	RF00885	scaffold2068	19891	20266	+	1.20E-26	microRNA MIR821
MIR821	RF00885	scaffold2399	2068	1932	-	0.00029	microRNA MIR821
MIR821	RF00885	scaffold2618	13061	13355	+	2.70E-13	microRNA MIR821
MIR821	RF00885	scaffold2618	13355	13061	-	4.30E-11	microRNA MIR821
MIR821	RF00885	scaffold2729	16153	16025	-	0.00051	microRNA MIR821
MIR821	RF00885	scaffold2863	6643	6743	+	0.01	microRNA MIR821

MIR821	RF00885	scaffold2893	13520	13640	+	0.00038	microRNA MIR821
MIR821	RF00885	scaffold3576	38035	37794	-	8.40E-06	microRNA MIR821
MIR821	RF00885	scaffold3913	7690	7842	+	5.80E-09	microRNA MIR821
MIR821	RF00885	scaffold8751	1028	773	-	1.80E-11	microRNA MIR821
MIR821	RF00885	scaffold9771	1	86	+	0.00019	microRNA MIR821
MIR821	RF00885	scaffold11036	2370	2479	+	1.50E-05	microRNA MIR821
MIR821	RF00885	C2451162	5	128	+	0.0012	microRNA MIR821
MIR821	RF00885	C2459249	4	130	+	2.30E-06	microRNA MIR821
MIR821	RF00885	C2492555	170	1	-	3.20E-10	microRNA MIR821
MIR821	RF00885	C2495423	1	174	+	0.00077	microRNA MIR821
MIR821	RF00885	C2498069	89	12	-	6.00E-07	microRNA MIR821
MIR821	RF00885	C2512957	1	193	+	2.70E-09	microRNA MIR821
MIR821	RF00885	C2512957	193	1	-	7.80E-07	microRNA MIR821
MIR821	RF00885	C2520529	3	212	+	2.00E-05	microRNA MIR821
MIR821	RF00885	C2524459	198	25	-	9.50E-19	microRNA MIR821
MIR821	RF00885	C2524459	29	194	+	2.40E-16	microRNA MIR821
MIR821	RF00885	C2531689	2	230	+	0.0018	microRNA MIR821
MIR821	RF00885	C2539219	209	1	-	0.0054	microRNA MIR821
MIR821	RF00885	C2539219	1	191	+	0.0058	microRNA MIR821
MIR821	RF00885	C2554962	184	58	-	0.0066	microRNA MIR821
MIR821	RF00885	C2556924	169	281	+	6.90E-05	microRNA MIR821
MIR821	RF00885	C2562204	60	202	+	0.0049	microRNA MIR821
MIR821	RF00885	C2563136	1	265	+	9.30E-09	microRNA MIR821
MIR821	RF00885	C2565696	182	90	-	2.20E-05	microRNA MIR821
MIR821	RF00885	C2569186	164	23	-	5.70E-06	microRNA MIR821
MIR821	RF00885	C2573744	292	32	-	7.70E-12	microRNA MIR821
MIR821	RF00885	C2592972	292	54	-	4.70E-08	microRNA MIR821
MIR845_2	RF00975	C2539605	60	193	+	6.50E-05	microRNA MIR845_2
MIR845_2	RF00975	C2539605	191	58	-	6.50E-05	microRNA MIR845_2
mir-85	RF00810	C2508861	87	159	+	0.0027	microRNA mir-85
mir-875	RF00933	C2443488	44	109	+	1.20E-05	microRNA mir-875
mir-875	RF00933	C2443488	106	43	-	0.0017	microRNA mir-875
mir-876	RF00935	scaffold3187	60305	60442	+	0.0077	microRNA mir-876
mir-883	RF00909	C2536359	67	141	+	3.50E-05	microRNA mir-883
mir-887	RF01035	scaffold699	3657	3736	+	0.0091	microRNA mir-887
mir-891	RF01042	C2428002	110	8	-	0.0013	microRNA mir-891

mir-90	RF00785	scaffold4055	634	715	+	3.10E-05	microRNA mir-90
mir-90	RF00785	scaffold4055	715	634	-	7.00E-05	microRNA mir-90
mir-90	RF00785	C2433444	11	94	+	3.70E-07	microRNA mir-90
mir-924	RF00999	C2456887	91	42	-	0.0061	microRNA mir-924
mir-927	RF00950	scaffold110	21540	21466	-	1.00E-12	microRNA mir-927
mir-927	RF00950	scaffold110	21466	21540	+	7.60E-05	microRNA mir-927
mir-927	RF00950	scaffold3915	736	806	+	0.0014	microRNA mir-927
mir-927	RF00950	C2501727	79	136	+	0.0006	microRNA mir-927
mir-927	RF00950	C2511849	122	192	+	0.0017	microRNA mir-927
mir-927	RF00950	C2532337	176	57	-	0.00026	microRNA mir-927
mir-929	RF00947	scaffold3837	1956	1858	-	3.50E-18	microRNA mir-929
mir-929	RF00947	scaffold3837	1867	1964	+	3.00E-07	microRNA mir-929
mir-932	RF01914	scaffold3171	7719	7624	-	8.30E-16	microRNA mir-932
mir-932	RF01914	scaffold3171	7634	7727	+	0.001	microRNA mir-932
mir-934	RF01025	C2490863	49	124	+	0.00017	microRNA mir-934
mir-944	RF01024	scaffold7148	3020	2945	-	1.20E-07	microRNA mir-944
mir-944	RF01024	C2412968	108	34	-	0.0081	microRNA mir-944
mir-96	RF00669	C2411576	103	24	-	0.0092	microRNA mir-96
mir-981	RF01926	scaffold248	22586	22697	+	7.50E-05	microRNA mir-981
mir-996	RF00948	scaffold940	117255	117155	-	0.00062	microRNA mir-996
mir-996	RF00948	scaffold4925	21802	21722	-	0.00068	microRNA mir-996
mir-iab-4	RF00725	scaffold7409	3707	3623	-	1.20E-20	microRNA mir-iab-4
mir-iab-4	RF00725	scaffold7409	3623	3707	+	2.10E-08	microRNA mir-iab-4
mir-1	RF00103	scaffold6180	16847	16920	+	1.50E-08	mir-1 microRNA precursor family
mir-1	RF00103	scaffold6180	16920	16847	-	0.0086	mir-1 microRNA precursor family
mir-1	RF00103	C2405622	11	80	+	0.0001	mir-1 microRNA precursor family
mir-1	RF00103	C2405622	80	11	-	0.00014	mir-1 microRNA precursor family
mir-10	RF00104	scaffold1830	312503	312573	+	2.40E-11	mir-10 microRNA precursor family
mir-10	RF00104	scaffold1830	367118	367047	-	1.20E-08	mir-10 microRNA precursor family
mir-10	RF00104	scaffold3112	52599	52672	+	3.70E-06	mir-10 microRNA precursor family
mir-10	RF00104	scaffold3112	52671	52598	-	0.0045	mir-10 microRNA precursor family
mir-10	RF00104	scaffold3474	42526	42456	-	7.70E-11	mir-10 microRNA precursor family
mir-10	RF00104	scaffold3474	42080	42153	+	7.10E-05	mir-10 microRNA precursor family
mir-10	RF00104	scaffold3474	42457	42527	+	0.00018	mir-10 microRNA precursor family
mir-122	RF00684	scaffold3208	2419	2476	+	0.00023	mir-122 microRNA precursor
mir-122	RF00684	scaffold3208	2470	2421	-	0.00047	mir-122 microRNA precursor

mir-122	RF00684	scaffold3435	333	406	+	6.60E-07	mir-122 microRNA precursor
mir-122	RF00684	scaffold5716	10250	10191	-	0.00036	mir-122 microRNA precursor
mir-122	RF00684	scaffold8295	135	81	-	3.50E-05	mir-122 microRNA precursor
mir-122	RF00684	scaffold8295	93	126	+	0.0049	mir-122 microRNA precursor
mir-122	RF00684	C2533709	139	206	+	1.20E-08	mir-122 microRNA precursor
mir-122	RF00684	C2588046	303	356	+	0.002	mir-122 microRNA precursor
mir-124	RF00239	scaffold6625	64258	64339	+	3.30E-11	mir-124 microRNA precursor family
mir-133	RF00446	scaffold6180	54104	54190	+	5.60E-13	mir-133 microRNA precursor family
mir-135	RF00246	C2433444	9	97	+	0.00039	mir-135 microRNA precursor family
mir-135	RF00246	C2459249	120	16	-	1.60E-05	mir-135 microRNA precursor family
mir-148	RF00248	C2539605	99	152	+	7.10E-07	mir-148/mir-152 microRNA precursor family
mir-148	RF00248	C2539605	152	99	-	7.10E-07	mir-148/mir-152 microRNA precursor family
mir-156	RF00073	C2449896	81	131	+	0.0079	mir-156 microRNA precursor
mir-166	RF00075	scaffold3843	4726	4660	-	0.0047	mir-166 microRNA precursor
mir-166	RF00075	scaffold4349	1887	2013	+	0.0071	mir-166 microRNA precursor
mir-166	RF00075	C2454758	1	134	+	6.80E-06	mir-166 microRNA precursor
mir-166	RF00075	C2454758	134	1	-	6.80E-06	mir-166 microRNA precursor
mir-17	RF00051	C2477855	4	71	+	0.0088	mir-17 microRNA precursor family
mir-172	RF00452	scaffold9212	12368	12541	+	0.006	mir-172 microRNA precursor family
mir-172	RF00452	C2415676	2	109	+	1.30E-07	mir-172 microRNA precursor family
mir-172	RF00452	C2415676	109	2	-	0.0019	mir-172 microRNA precursor family
mir-172	RF00452	C2441632	110	17	-	0.0043	mir-172 microRNA precursor family
mir-172	RF00452	C2448856	15	116	+	0.0077	mir-172 microRNA precursor family
mir-172	RF00452	C2448856	116	15	-	0.008	mir-172 microRNA precursor family
mir-172	RF00452	C2454758	14	115	+	0.00065	mir-172 microRNA precursor family
mir-172	RF00452	C2454758	121	20	-	0.00065	mir-172 microRNA precursor family
mir-172	RF00452	C2459249	25	110	+	0.0013	mir-172 microRNA precursor family
mir-172	RF00452	C2492555	34	137	+	8.50E-07	mir-172 microRNA precursor family
mir-172	RF00452	C2492555	137	34	-	0.00014	mir-172 microRNA precursor family
mir-172	RF00452	C2528141	28	105	+	2.20E-05	mir-172 microRNA precursor family
mir-172	RF00452	C2532337	58	175	+	0.00086	mir-172 microRNA precursor family
mir-181	RF00076	C2554962	152	86	-	0.0085	mir-181 microRNA precursor
mir-19	RF00245	C2405936	12	60	+	0.0011	mir-19 microRNA precursor family
mir-192	RF00130	scaffold2266	1563	1605	+	0.0098	mir-192/215 microRNA precursor
mir-192	RF00130	scaffold5344	1122	1182	+	0.0026	mir-192/215 microRNA precursor
mir-194	RF00257	scaffold3210	942	1092	+	0.0013	mir-194 microRNA precursor family

mir-194	RF00257	scaffold3210	1091	941	-	0.0026	mir-194 microRNA precursor family
mir-196	RF00256	C2398502	1	77	+	0.005	mir-196 microRNA precursor family
mir-2	RF00047	scaffold3143	15255	15322	+	1.50E-10	mir-2 microRNA precursor
mir-2	RF00047	scaffold3143	14980	15048	+	9.10E-08	mir-2 microRNA precursor
mir-2	RF00047	scaffold3143	15397	15464	+	1.20E-06	mir-2 microRNA precursor
mir-2	RF00047	scaffold3143	15124	15188	+	5.50E-06	mir-2 microRNA precursor
mir-2	RF00047	scaffold3143	15320	15253	-	0.00051	mir-2 microRNA precursor
mir-2	RF00047	scaffold3143	15046	14978	-	0.0023	mir-2 microRNA precursor
mir-2	RF00047	scaffold3143	15186	15122	-	0.0024	mir-2 microRNA precursor
mir-218	RF00255	C2538405	148	206	+	3.10E-05	mir-218 microRNA precursor family
mir-219	RF00251	scaffold3884	44770	44836	+	1.40E-07	mir-219 microRNA precursor family
mir-219	RF00251	scaffold4308	412	479	+	0.0087	mir-219 microRNA precursor family
mir-24	RF00178	C2525777	126	161	+	0.0021	mir-24 microRNA precursor family
mir-29	RF00074	scaffold455	11553	11617	+	6.00E-05	mir-29 microRNA precursor
mir-2985-2	RF02095	C2448856	105	26	-	0.0061	mir-2985-2 microRNA precursor
mir-2985-2	RF02095	C2533709	207	140	-	0.00019	mir-2985-2 microRNA precursor
mir-2985-2	RF02095	C2533709	131	204	+	0.00033	mir-2985-2 microRNA precursor
mir-34	RF00456	C2498069	89	12	-	1.70E-06	mir-34 microRNA precursor family
mir-34	RF00456	C2525421	75	150	+	0.0085	mir-34 microRNA precursor family
mir-395	RF00451	C2390636	4	99	+	5.50E-06	mir-395 microRNA precursor family
mir-395	RF00451	C2448856	116	15	-	3.00E-07	mir-395 microRNA precursor family
mir-395	RF00451	C2451162	19	114	+	8.40E-06	mir-395 microRNA precursor family
mir-395	RF00451	C2451162	114	19	-	6.40E-05	mir-395 microRNA precursor family
mir-46	RF00249	scaffold1538	57195	57130	-	7.80E-09	mir-46/mir-47/mir-281 microRNA precursor far
mir-6	RF00143	scaffold6995	60	125	+	0.00049	mir-6 microRNA precursor
mir-7	RF00053	scaffold3135	9569	9654	+	2.50E-08	mir-7 microRNA precursor
mir-8	RF00241	scaffold788	126471	126394	-	2.40E-12	mir-8/mir-141/mir-200 microRNA precursor far
mir-8	RF00241	scaffold788	126394	126470	+	1.70E-05	mir-8/mir-141/mir-200 microRNA precursor far
mir-9	RF00237	scaffold519	33789	33730	-	2.40E-10	mir-9/mir-79 microRNA precursor family
mir-9	RF00237	scaffold519	33730	33789	+	0.0084	mir-9/mir-79 microRNA precursor family
mir-9	RF00237	scaffold782	25664	25725	+	0.00079	mir-9/mir-79 microRNA precursor family
mir-9	RF00237	scaffold1448	94508	94567	+	2.90E-15	mir-9/mir-79 microRNA precursor family
mir-9	RF00237	scaffold1448	94567	94508	-	8.20E-06	mir-9/mir-79 microRNA precursor family
mir-9	RF00237	scaffold1580	274618	274679	+	0.00017	mir-9/mir-79 microRNA precursor family
mir-9	RF00237	scaffold1580	275341	275402	+	0.0056	mir-9/mir-79 microRNA precursor family
mir-9	RF00237	scaffold4347	3076	3136	+	0.0068	mir-9/mir-79 microRNA precursor family

mir-9	RF00237	scaffold9030	3230	3289	+	7.80E-06	mir-9/mir-79 microRNA precursor family
mir-9	RF00237	C2398502	1	63	+	9.80E-06	mir-9/mir-79 microRNA precursor family
mir-9	RF00237	C2398502	63	1	-	0.00012	mir-9/mir-79 microRNA precursor family
mir-92	RF00464	scaffold1707	48009	47933	-	0.0082	mir-92 microRNA precursor family
mir-92	RF00464	scaffold2503	36146	36074	-	0.0077	mir-92 microRNA precursor family
mir-BHRF1-2	RF00366	scaffold7148	3018	2947	-	6.80E-06	mir-BHRF1-2 microRNA precursor family
mir-BHRF1-2	RF00366	C2512027	52	121	+	0.0059	mir-BHRF1-2 microRNA precursor family
mir-BHRF1-2	RF00366	C2554962	148	90	-	0.0039	mir-BHRF1-2 microRNA precursor family
mycoplasma_	RF01842	scaffold7103	612	527	-	0.009	mycoplasma ribosomal frameshift element
NRON	RF00636	C2547964	1	71	+	0.0089	ncRNA Repressor of NFAT (nuclear factor of act
neisseria_FSE	RF01843	C2511391	93	60	-	0.0036	neiserria ribosomal frameshift element
NEAT1_2	RF01956	scaffold8575	1179	1077	-	0.002	Nuclear enriched abundant transcript 1 conserv
RNaseP_nuc	RF00009	scaffold2233	67284	66987	-	4.60E-43	Nuclear RNase P
OLE	RF01071	C2424304	114	62	-	0.0091	Ornate Large Extremophilic RNA
IRES_Pesti	RF00209	scaffold3736	5449	5596	+	0.00016	Pestivirus internal ribosome entry site (IRES)
Phe_leader	RF01859	scaffold2187	4039	4160	+	0.0062	Phenylalanine leader peptide
Phe_leader	RF01859	scaffold9190	107	7	-	0.0037	Phenylalanine leader peptide
Phe_leader	RF01859	C2400106	96	1	-	5.40E-05	Phenylalanine leader peptide
Phe_leader	RF01859	C2400106	1	95	+	0.0021	Phenylalanine leader peptide
Phe_leader	RF01859	C2405622	94	1	-	0.00096	Phenylalanine leader peptide
Phe_leader	RF01859	C2482023	19	136	+	0.0033	Phenylalanine leader peptide
Phe_leader	RF01859	C2485491	63	163	+	0.00061	Phenylalanine leader peptide
Plant_SRP	RF01855	scaffold326	109712	109418	-	1.50E-21	Plant signal recognition particle RNA
Plant_U3	RF01847	scaffold2237	58533	58660	+	2.00E-06	Plant small nucleolar RNA U3
Plant_U3	RF01847	scaffold7441	1806	1767	-	0.0068	Plant small nucleolar RNA U3
Plant_U3	RF01847	scaffold10074	5652	5502	-	1.60E-10	Plant small nucleolar RNA U3
RUF2	RF01579	C2405622	6	105	+	0.0098	Plasmodium RNA of unknown function RUF2
RUF1	RF01578	C2494891	173	97	-	0.0088	Plasmodium RNA of unkown function RUF1
K_chan_RES	RF00485	scaffold654	21341	21454	+	1.40E-18	Potassium channel RNA editing signal
K_chan_RES	RF00485	scaffold1121	38879	38767	-	2.40E-13	Potassium channel RNA editing signal
K_chan_RES	RF00485	scaffold1502	46089	45984	-	1.20E-06	Potassium channel RNA editing signal
K_chan_RES	RF00485	scaffold2828	45721	45608	-	8.00E-10	Potassium channel RNA editing signal
K_chan_RES	RF00485	scaffold5773	5323	5436	+	1.20E-20	Potassium channel RNA editing signal
Protozoa_SRI	RF01856	scaffold326	109661	109531	-	6.20E-07	Protozoan signal recognition particle RNA
PK-G12rRNA	RF01118	C2663579	387	494	+	4.20E-32	Pseudoknot of the domain G(G12) of 23S ribos
CrcZ	RF01675	scaffold10463	9066	8998	-	0.0077	Pseudomonas sRNA CrcZ

P10	RF01668	C2612118	98	168	+	0.0025	Pseudomonas sRNA P10
P11	RF00625	scaffold4379	2260	2133	-	0.0079	Pseudomonas sRNA P11
P8	RF01682	C2515883	123	51	-	0.0012	Pseudomonas sRNA P8
Purine	RF00167	scaffold2591	29783	29884	+	4.00E-15	Purine riboswitch
Purine	RF00167	C2553084	143	238	+	0.0088	Purine riboswitch
PyrR	RF00515	scaffold4462	101670	101541	-	0.0041	PyrR binding site
R2_retro_el	RF00524	C2390178	19	96	+	0.0044	R2 RNA element
REN-SRE	RF00180	C2531639	120	84	-	0.0031	Renin stability regulatory element (REN-SRE)
RMST_10	RF01971	scaffold5490	9126	8984	-	0.0023	Rhabdomyosarcoma 2 associated transcript co
RMST_9	RF01970	scaffold6	52405	52591	+	0.0019	Rhabdomyosarcoma 2 associated transcript co
RMST_9	RF01970	scaffold73	65254	65447	+	0.0043	Rhabdomyosarcoma 2 associated transcript co
L10_leader	RF00557	scaffold5346	17786	17637	-	7.50E-10	Ribosomal protein L10 leader
L20_leader	RF00558	scaffold5295	28897	28755	-	0.00063	Ribosomal protein L20 leader
L21_leader	RF00559	scaffold4462	81554	81636	+	8.00E-07	Ribosomal protein L21 leader
RUF20	RF01824	C2397138	54	101	+	0.0024	RNA of unknown function 20
RUF21	RF01825	C2404272	73	1	-	0.0052	RNA of unknown function 21
RUF21	RF01825	C2420536	1	62	+	0.0027	RNA of unknown function 21
RUF4	RF01582	C2405622	81	1	-	0.009	RNA of unknown function RUF4
RUF4	RF01582	C2454322	134	11	-	0.00014	RNA of unknown function RUF4
RsaJ	RF01822	C2430464	3	118	+	0.0048	RNA Staph. aureus A
RsaJ	RF01822	C2486207	109	158	+	0.0015	RNA Staph. aureus A
RsaD	RF01819	C2500331	180	123	-	0.0048	RNA Staph. aureus D
RNA-OUT	RF00240	C2395444	1	48	+	4.10E-11	RNA-OUT
rne5	RF00040	C2492745	89	121	+	0.0097	RNase E 5' UTR element
RNase_MRP	RF00030	scaffold972	7385	7285	-	0.00065	RNase MRP
RybB	RF00110	C2585656	137	62	-	0.007	RybB RNA
STnc300	RF02070	scaffold2301	42035	41899	-	0.0082	Salmonella enterica sRNA STnc300
STnc470	RF02050	C2596462	110	1	-	0.0039	Salmonella enterica sRNA STnc470
SAM	RF00162	scaffold9019	49416	49329	-	2.40E-15	SAM riboswitch (S box leader)
SAM_V	RF01826	scaffold8344	14733	14668	-	7.80E-05	SAM-V riboswitch
SMAD5-AS1_	RF02173	C2393508	101	68	-	0.0017	SMAD5 antisense RNA 1 conserved region 1
SCARNA8	RF00286	scaffold5858	20670	20804	+	5.40E-08	Small Cajal body specific RNA 8
SCARNA7	RF01295	C2575116	81	231	+	0.0089	Small Cajal body-specific RNA 7
snoMe28S-A	RF00535	scaffold4175	2639	2716	+	7.00E-07	Small nucleolar RNA Me28S-Am982
snopsi28S-11	RF00542	scaffold2846	40868	40791	-	0.00087	Small nucleolar RNA psi28S-1192
snoR160	RF00203	C2488637	77	7	-	0.0012	Small nucleolar RNA R160

snoR43	RF00316	C2493281	63	17	-	0.0051	Small nucleolar RNA R43
sn2524	RF01212	C2447290	100	3	-	0.005	Small nucleolar RNA sn2524
sn2991	RF01202	C2646725	667	570	-	0.0057	Small nucleolar RNA sn2991
snoR100	RF01219	C2480621	118	5	-	0.002	Small nucleolar RNA snoR100
snoR101	RF01155	scaffold5736	10870	10811	-	0.0031	Small nucleolar RNA snoR101
snoR101	RF01155	C2408526	105	83	-	0.0091	Small nucleolar RNA snoR101
snoR101	RF01155	C2508919	109	62	-	0.0039	Small nucleolar RNA snoR101
snoR101	RF01155	C2508919	77	123	+	0.0057	Small nucleolar RNA snoR101
snoR104	RF01220	C2427540	22	79	+	0.009	Small nucleolar RNA snoR104
snoR113	RF01420	C2422908	33	75	+	0.0075	Small nucleolar RNA snoR113
snoR116	RF01422	C2512661	27	106	+	0.0017	small nucleolar RNA snoR116
snoR118	RF01424	scaffold1718	5837	5907	+	0.00039	small nucleolar RNA snoR118
snoR137	RF01433	scaffold4928	3835	3939	+	0.0055	small nucleolar RNA snoR137
snoR18	RF01594	scaffold1971	6918	6863	-	0.0056	small nucleolar RNA snoR18
plasmodium_	RF01596	C2412466	25	68	+	0.0057	small nucleolar RNA snoR20
snoR4	RF01171	C2433836	6	47	+	0.0096	Small nucleolar RNA snoR4
snoR4	RF01171	C2473333	69	6	-	0.0023	Small nucleolar RNA snoR4
snoR4	RF01171	C2540426	244	182	-	0.00075	Small nucleolar RNA snoR4
snoR4	RF01171	C2568198	152	181	+	0.0083	Small nucleolar RNA snoR4
snoR4	RF01171	C2603130	384	424	+	0.0045	Small nucleolar RNA snoR4
snoR639	RF00291	scaffold3760	17573	17706	+	2.60E-05	Small nucleolar RNA snoR639/H1
snoR64a	RF01163	scaffold2374	12899	12962	+	0.0081	Small nucleolar RNA snoR64a
snoR64a	RF01163	C2440924	67	10	-	0.0083	Small nucleolar RNA snoR64a
snoR64a	RF01163	C2534177	188	142	-	0.002	Small nucleolar RNA snoR64a
snoR64a	RF01163	C2584024	252	320	+	0.0087	Small nucleolar RNA snoR64a
SNORA13	RF00396	scaffold2193	63496	63569	+	0.0023	Small nucleolar RNA SNORA13
SNORA16	RF00190	C2458381	39	129	+	0.0086	Small nucleolar RNA SNORA16B/SNORA16A far
SNORA3	RF00334	scaffold1923	81462	81380	-	0.0024	Small nucleolar RNA SNORA3/SNORA45 family
SNORA44	RF00405	C2629647	347	476	+	0.0071	Small nucleolar RNA SNORA44
SNORA49	RF00562	scaffold4235	2494	2416	-	0.0077	Small nucleolar RNA SNORA49
SNORA57	RF00191	scaffold5855	53025	52888	-	0.00068	Small nucleolar RNA SNORA57
SNORA62	RF00091	scaffold940	47550	47697	+	0.0043	Small nucleolar RNA SNORA62/SNORA6 family
SNORA71	RF00056	C2501727	57	172	+	0.004	Small nucleolar RNA SNORA71
SNORA73	RF00045	scaffold1844	36367	36189	-	1.50E-07	Small nucleolar RNA SNORA73 family
SNORA74	RF00090	scaffold10877	6652	6458	-	1.50E-08	Small nucleolar RNA SNORA74
SNORD100	RF00609	C2561740	227	303	+	0.00052	Small nucleolar RNA SNORD100

SNORD18	RF00093	C2393664	11	90	+	0.0069	Small nucleolar RNA SNORD18
SNORD2	RF01299	C2527153	173	109	-	0.0048	Small nucleolar RNA SNORD2
SNORD27	RF00086	scaffold2341	15460	15416	-	0.0084	Small nucleolar RNA SNORD27
SNORD31	RF00089	scaffold3760	11282	11353	+	3.80E-08	Small nucleolar RNA SNORD31
SNORD31	RF00089	scaffold3760	15051	15123	+	5.60E-07	Small nucleolar RNA SNORD31
SNORD31	RF00089	scaffold3760	14798	14870	+	0.00015	Small nucleolar RNA SNORD31
SNORD31	RF00089	C2497331	67	126	+	0.00043	Small nucleolar RNA SNORD31
SNORD5	RF01161	scaffold1034	36	75	+	0.0086	Small nucleolar RNA SNORD5
SNORD5	RF01161	scaffold2784	1502	1456	-	0.0083	Small nucleolar RNA SNORD5
SNORD5	RF01161	C2478419	46	4	-	0.00032	Small nucleolar RNA SNORD5
SNORD5	RF01161	C2516429	89	43	-	0.0014	Small nucleolar RNA SNORD5
SNORD5	RF01161	C2583572	240	190	-	0.0028	Small nucleolar RNA SNORD5
SNORD57	RF00274	scaffold4545	49531	49470	-	0.0076	Small nucleolar RNA SNORD57
SNORD65	RF00571	scaffold2247	5476	5432	-	0.0076	Small nucleolar RNA SNORD65
SNORD77	RF00591	scaffold1756	21670	21613	-	0.0013	Small nucleolar RNA SNORD77
SNORD78	RF00592	scaffold1480	6842	6795	-	0.0012	Small nucleolar RNA SNORD78
SNORD78	RF00592	C2485749	113	79	-	0.0065	Small nucleolar RNA SNORD78
SNORD78	RF00592	C2485955	113	79	-	0.0057	Small nucleolar RNA SNORD78
snR40	RF01201	scaffold370	675	752	+	0.00013	Small nucleolar RNA snR40
snR45	RF01266	scaffold1659	36914	36788	-	0.0018	Small nucleolar RNA snR45
snR45	RF01266	C2390598	92	18	-	0.0051	Small nucleolar RNA snR45
snR52	RF01195	C2496093	60	124	+	0.0019	Small nucleolar RNA snR52
snosnR54	RF00473	C2390142	73	40	-	0.009	Small nucleolar RNA snR54
snosnR54	RF00473	C2406850	29	72	+	0.0034	Small nucleolar RNA snR54
snosnR54	RF00473	C2406912	93	20	-	0.0066	Small nucleolar RNA snR54
snosnR55	RF00472	C2522173	81	151	+	0.0016	Small nucleolar RNA snR55/Z10
snosnR55	RF00472	C2525875	219	152	-	0.0092	Small nucleolar RNA snR55/Z10
snosnR55	RF00472	C2530763	86	37	-	0.0075	Small nucleolar RNA snR55/Z10
snR58	RF01199	scaffold8780	762	832	+	0.0015	Small nucleolar RNA snR58
snosnR60_Z1	RF00309	scaffold4960	4300	4386	+	0.00014	Small nucleolar RNA snR60/Z15/Z230/Z193/J17
snosnR61	RF00476	scaffold544	32623	32567	-	0.00039	Small nucleolar RNA snR61/Z1/Z11
snosnR61	RF00476	scaffold2157	43131	43065	-	8.50E-08	Small nucleolar RNA snR61/Z1/Z11
snosnR61	RF00476	scaffold2157	42651	42598	-	1.10E-07	Small nucleolar RNA snR61/Z1/Z11
snosnR61	RF00476	scaffold6382	72124	72053	-	0.0056	Small nucleolar RNA snR61/Z1/Z11
snosnR61	RF00476	C2402764	58	3	-	0.00061	Small nucleolar RNA snR61/Z1/Z11
snosnR61	RF00476	C2418382	17	72	+	0.0042	Small nucleolar RNA snR61/Z1/Z11

snosnR61	RF00476	C2420490	27	70	+	0.0024	Small nucleolar RNA snR61/Z1/Z11
snosnR61	RF00476	C2445242	77	27	-	0.0057	Small nucleolar RNA snR61/Z1/Z11
snosnR61	RF00476	C2464401	106	55	-	0.0016	Small nucleolar RNA snR61/Z1/Z11
snosnR61	RF00476	C2474243	83	133	+	0.0048	Small nucleolar RNA snR61/Z1/Z11
snR65	RF01204	scaffold2097	1294	1257	-	0.0047	Small nucleolar RNA snR65
snR65	RF01204	scaffold7751	4812	4892	+	0.0028	Small nucleolar RNA snR65
snR65	RF01204	scaffold8185	7036	6957	-	0.0043	Small nucleolar RNA snR65
snR67	RF01177	C2546262	101	160	+	0.0012	Small nucleolar RNA snR67
snR73	RF01207	scaffold26	41891	41992	+	0.0097	Small nucleolar RNA snR73
snR73	RF01207	C2398154	93	15	-	0.00021	Small nucleolar RNA snR73
snR73	RF01207	C2454418	72	10	-	0.0077	Small nucleolar RNA snR73
snR75	RF01185	C2443186	96	34	-	0.0012	Small nucleolar RNA snR75
snR77	RF01181	scaffold6431	2336	2393	+	0.0036	Small nucleolar RNA snR77
snR77	RF01181	scaffold9187	2440	2374	-	0.0039	Small nucleolar RNA snR77
snR77	RF01181	C2533851	169	110	-	0.0039	Small nucleolar RNA snR77
snR77	RF01181	C2591114	346	398	+	0.0061	Small nucleolar RNA snR77
snR82	RF01261	scaffold8142	1208	1330	+	0.0089	Small nucleolar RNA snR82
S_pombe_sn	RF01447	C2402970	103	42	-	0.0032	small nucleolar RNA snR98
sR11	RF01150	C2418656	55	93	+	0.0011	Small nucleolar RNA sR11
sR15	RF01146	C2430664	98	47	-	0.007	Small nucleolar RNA sR15
sR2	RF01139	C2398332	90	51	-	0.008	Small nucleolar RNA sR2
sR2	RF01139	C2444538	82	49	-	0.0069	Small nucleolar RNA sR2
sR2	RF01139	C2576314	279	241	-	0.0037	Small nucleolar RNA sR2
sR21	RF01137	C2529275	1	51	+	0.0049	Small nucleolar RNA sR21
sR38	RF01121	C2393986	95	62	-	0.0006	Small nucleolar RNA sR38
sR41	RF01126	C2461049	60	25	-	0.009	Small nucleolar RNA sR41
sR52	RF01306	C2396848	55	97	+	0.0044	Small nucleolar RNA sR52
sR52	RF01306	C2450054	72	128	+	0.0036	Small nucleolar RNA sR52
sR53	RF01276	C2492255	41	22	-	0.0023	Small nucleolar RNA sR53
snoU105B	RF01173	scaffold2640	1023	1083	+	0.0014	Small nucleolar RNA U105B
snoU18	RF01159	C2406912	96	55	-	0.0073	Small nucleolar RNA U18
snoU2-30	RF00493	scaffold7262	3112	3171	+	0.0041	Small nucleolar RNA U2-30
U3	RF00012	scaffold2237	58534	58660	+	1.00E-07	Small nucleolar RNA U3
U3	RF00012	scaffold10074	5653	5502	-	3.30E-13	Small nucleolar RNA U3
snoU85	RF01296	scaffold6094	3717	3664	-	0.0017	Small nucleolar RNA U85
snoZ122	RF00343	C2493849	87	148	+	0.0013	Small nucleolar RNA Z122

snoZ221_sno	RF00300	scaffold2898	98603	98530	-	0.0035	Small nucleolar RNA Z221/R21b
snoZ267	RF00344	scaffold1211	2617	2690	+	0.0073	Small nucleolar RNA Z267
snoZ267	RF00344	C2620629	355	427	+	0.00027	Small nucleolar RNA Z267
snoZ39	RF00341	scaffold7009	1639	1593	-	0.0049	Small nucleolar RNA Z39
snoZ5	RF01226	scaffold1030	10227	10285	+	0.0036	Small nucleolar RNA Z5
Afu_199	RF01501	C2423540	56	14	-	0.0083	snoRNA A. fimigatus snoRNA Afu_199
Afu_199	RF01501	C2517287	77	41	-	0.0074	snoRNA A. fimigatus snoRNA Afu_199
Afu_298	RF01507	scaffold3180	25039	24966	-	0.0055	snoRNA A. fimigatus snoRNA Afu_298
Afu_298	RF01507	scaffold6388	621	685	+	0.0045	snoRNA A. fimigatus snoRNA Afu_298
Afu_298	RF01507	C2410064	27	83	+	0.0043	snoRNA A. fimigatus snoRNA Afu_298
Afu_298	RF01507	C2707083	2838	2895	+	0.0003	snoRNA A. fimigatus snoRNA Afu_298
Afu_190	RF01498	scaffold10282	685	639	-	0.0073	snoRNA A. fumigatus snoRNA Afu_190
Afu_191	RF01499	C2471415	94	42	-	0.004	snoRNA A. fumigatus snoRNA Afu_191
Afu_198	RF01500	C2604890	306	346	+	0.0044	snoRNA A. fumigatus snoRNA Afu_198
Afu_264	RF01505	C2485519	28	65	+	0.0054	snoRNA A. fumigatus snoRNA Afu_264
Afu_264	RF01505	scaffold8860	2760	2839	+	0.0028	snoRNA A. fumigatus snoRNA Afu_264
Afu_304	RF01511	C2553238	272	207	-	0.0083	snoRNA A. fumigatus snoRNA Afu_304
Afu_335	RF01513	C2548280	160	217	+	0.0039	snoRNA A. fumigatus snoRNA Afu_335
Afu_455	RF01860	C2398660	92	35	-	0.0068	snoRNA A. fumigatus snoRNA Afu_455
Afu_455	RF01860	C2544618	177	220	+	0.0082	snoRNA A. fumigatus snoRNA Afu_455
Afu_514	RF01515	scaffold2639	28311	28235	-	0.003	snoRNA A. fumigatus snoRNA Afu_514
ceN100	RF01607	scaffold1923	81463	81330	-	0.00035	snoRNA C. elegans snoRNA ceN100
ceN103	RF01609	C2627893	332	226	-	0.0036	snoRNA C. elegans snoRNA ceN103
ceN28	RF01625	C2411754	97	18	-	0.0036	snoRNA C. elegans snoRNA ceN28
ceN28	RF01625	C2390554	62	100	+	0.0041	snoRNA C. elegans snoRNA ceN28
ceN53	RF01641	scaffold7135	4951	4882	-	0.00026	snoRNA C. elegans snoRNA ceN53
ceN84	RF01655	scaffold1923	82500	82362	-	1.20E-06	snoRNA C. elegans snoRNA ceN84
DdR7	RF01575	scaffold10917	4302	4255	-	0.0062	snoRNA D. discoideum snoRNA Dd7
DdR10	RF01560	C2391978	94	44	-	0.0084	snoRNA D. discoideum snoRNA DdR10
DdR11	RF01561	scaffold5292	1803	1862	+	0.0066	snoRNA D. discoideum snoRNA DdR11
DdR11	RF01561	scaffold8995	511	574	+	0.0054	snoRNA D. discoideum snoRNA DdR11
DdR11	RF01561	C2423592	24	50	+	0.0054	snoRNA D. discoideum snoRNA DdR11
DdR12	RF01562	scaffold849	10553	10514	-	0.0084	snoRNA D. discoideum snoRNA DdR12
DdR12	RF01562	C2436930	91	49	-	0.0015	snoRNA D. discoideum snoRNA DdR12
DdR12	RF01562	C2460321	126	76	-	0.0028	snoRNA D. discoideum snoRNA DdR12
DdR13	RF01563	C2555294	114	62	-	0.0034	snoRNA D. discoideum snoRNA DdR13

DdR14	RF01564	scaffold481	11021	11066	+	0.0033	snoRNA D. discoideum snoRNA DdR14
DdR14	RF01564	C2561862	133	79	-	0.0051	snoRNA D. discoideum snoRNA DdR14
DdR15	RF01565	scaffold3110	5626	5688	+	0.0051	snoRNA D. discoideum snoRNA DdR15
DdR15	RF01565	scaffold4801	362	288	-	0.0035	snoRNA D. discoideum snoRNA DdR15
DdR15	RF01565	C2396174	62	98	+	0.0076	snoRNA D. discoideum snoRNA DdR15
DdR16	RF01566	C2432876	58	7	-	0.00032	snoRNA D. discoideum snoRNA DdR16
DdR16	RF01566	C2498281	31	79	+	0.00091	snoRNA D. discoideum snoRNA DdR16
DdR16	RF01566	C2525933	116	146	+	0.0015	snoRNA D. discoideum snoRNA DdR16
DdR16	RF01566	C2551528	161	213	+	0.0095	snoRNA D. discoideum snoRNA DdR16
DdR17	RF01567	C2394590	54	16	-	0.0053	snoRNA D. discoideum snoRNA DdR17
DdR17	RF01567	C2493211	134	98	-	0.0097	snoRNA D. discoideum snoRNA DdR17
DdR17	RF01567	C2516451	200	154	-	0.0036	snoRNA D. discoideum snoRNA DdR17
DdR2	RF01569	scaffold326	81172	81108	-	0.0033	snoRNA D. discoideum snoRNA DdR2
DdR2	RF01569	scaffold1397	15531	15580	+	0.0041	snoRNA D. discoideum snoRNA DdR2
DdR2	RF01569	scaffold5539	17452	17404	-	0.0034	snoRNA D. discoideum snoRNA DdR2
DdR2	RF01569	C2398564	80	10	-	0.0011	snoRNA D. discoideum snoRNA DdR2
DdR2	RF01569	C2439064	13	50	+	0.0035	snoRNA D. discoideum snoRNA DdR2
DdR4	RF01572	scaffold4741	5728	5682	-	0.0092	snoRNA D. discoideum snoRNA DdR4
DdR4	RF01572	C2467537	56	21	-	0.00043	snoRNA D. discoideum snoRNA DdR4
DdR5	RF01573	scaffold5187	382	336	-	0.0061	snoRNA D. discoideum snoRNA DdR5
DdR5	RF01573	scaffold6270	1784	1730	-	0.0031	snoRNA D. discoideum snoRNA DdR5
DdR5	RF01573	scaffold8347	8844	8909	+	0.0059	snoRNA D. discoideum snoRNA DdR5
DdR5	RF01573	scaffold10592	199	131	-	0.0029	snoRNA D. discoideum snoRNA DdR5
DdR5	RF01573	scaffold11118	1197	1141	-	0.0033	snoRNA D. discoideum snoRNA DdR5
DdR5	RF01573	C2445666	55	7	-	0.0061	snoRNA D. discoideum snoRNA DdR5
DdR5	RF01573	C2451178	123	83	-	0.0012	snoRNA D. discoideum snoRNA DdR5
DdR5	RF01573	C2515929	119	160	+	0.002	snoRNA D. discoideum snoRNA DdR5
DdR5	RF01573	C2606456	32	75	+	0.0015	snoRNA D. discoideum snoRNA DdR5
DdR6	RF01574	scaffold5075	11845	11803	-	0.0018	snoRNA D. discoideum snoRNA DdR6
DdR6	RF01574	C2399364	22	49	+	0.0091	snoRNA D. discoideum snoRNA DdR6
DdR6	RF01574	C2475267	127	87	-	0.0024	snoRNA D. discoideum snoRNA DdR6
sR6	RF01829	C2437080	75	119	+	0.003	snoRNA sR6
TB11Cs5H2	RF01543	scaffold466	181356	181289	-	0.0003	snoRNA Trypanosomatid snoRNA TB11Cs5H2
U8	RF00096	scaffold2342	20296	20177	-	0.00095	snoRNA U8 small nucleolar RNA
SOX2OT_exo	RF01952	C2473081	45	105	+	0.009	SOX2 overlapping transcript exon 2
speF	RF00518	C2622761	386	503	+	0.0055	speF leader

STnc290	RF01403	C2445704	69	116	+	0.0015	STnc290 Hfq binding RNA
STnc490k	RF01405	scaffold3211	10068	10127	+	0.0097	STnc490k Hfq binding RNA
sucC	RF01759	scaffold1279	12027	11960	-	0.0064	sucC RNA
suhB	RF00519	scaffold4977	521	577	+	0.0013	suhB
suhB	RF00519	C2391754	84	32	-	0.0083	suhB
suhB	RF00519	C2439192	104	27	-	0.0073	suhB
Tombus_5	RF00171	C2522085	215	126	-	0.0026	Tombusvirus 5' UTR
TPP	RF00059	scaffold8667	20426	20316	-	2.30E-14	TPP riboswitch (THI element)
traJ_5	RF00243	scaffold1040	877	972	+	0.008	traJ 5' UTR
traJ-II	RF01760	C2552622	219	283	+	0.0084	traJ-II RNA
tmRNA	RF00023	scaffold9788	4413	4488	+	1.10E-07	transfer-messenger RNA
tmRNA	RF00023	scaffold10187	17470	17116	-	7.10E-68	transfer-messenger RNA
tfoR	RF02100	scaffold1561	6548	6461	-	0.0051	Translational regulator of tfoXVC
tRNA	RF00005	scaffold64	32191	32119	-	1.10E-13	tRNA
tRNA	RF00005	scaffold98	85594	85523	-	2.60E-12	tRNA
tRNA	RF00005	scaffold1187	76322	76243	-	1.20E-09	tRNA
tRNA	RF00005	scaffold2166	64786	64857	+	6.40E-08	tRNA
tRNA	RF00005	scaffold3318	41609	41681	+	1.50E-13	tRNA
tRNA	RF00005	scaffold3488	50294	50385	+	2.30E-08	tRNA
tRNA	RF00005	scaffold3900	72752	72828	+	0.00015	tRNA
tRNA	RF00005	scaffold4116	4242	4172	-	2.10E-11	tRNA
tRNA	RF00005	scaffold5346	65200	65130	-	2.00E-11	tRNA
tRNA	RF00005	scaffold5971	26162	26090	-	0.0046	tRNA
tRNA	RF00005	scaffold6077	83631	83712	+	1.70E-11	tRNA
tRNA	RF00005	scaffold6758	26769	26678	-	2.90E-09	tRNA
tRNA	RF00005	scaffold6925	65407	65337	-	7.30E-12	tRNA
tRNA	RF00005	scaffold6925	9818	9891	+	8.50E-12	tRNA
tRNA	RF00005	scaffold7161	18354	18427	+	7.40E-13	tRNA
tRNA	RF00005	scaffold7868	2728	2656	-	5.90E-17	tRNA
tRNA	RF00005	scaffold7868	2119	2046	-	4.10E-15	tRNA
tRNA	RF00005	scaffold7868	2812	2741	-	9.40E-14	tRNA
tRNA	RF00005	scaffold7868	2498	2425	-	1.20E-13	tRNA
tRNA	RF00005	scaffold7868	2405	2332	-	2.10E-13	tRNA
tRNA	RF00005	scaffold7868	2906	2825	-	5.00E-12	tRNA
tRNA	RF00005	scaffold7868	2604	2520	-	1.70E-10	tRNA
tRNA	RF00005	scaffold7868	2212	2140	-	1.10E-07	tRNA

tRNA	RF00005	scaffold8804	49713	49605	-	7.70E-10	tRNA
tRNA	RF00005	scaffold9123	25860	25948	+	8.40E-09	tRNA
tRNA	RF00005	scaffold9123	25735	25818	+	1.40E-07	tRNA
tRNA	RF00005	scaffold9445	3418	3335	-	7.20E-09	tRNA
tRNA	RF00005	scaffold9525	9759	9831	+	6.50E-16	tRNA
tRNA	RF00005	scaffold9525	9655	9727	+	1.10E-10	tRNA
tRNA	RF00005	scaffold9525	9843	9923	+	1.60E-09	tRNA
tRNA	RF00005	scaffold9788	4413	4485	+	1.20E-11	tRNA
tRNA	RF00005	scaffold9788	4321	4408	+	9.50E-10	tRNA
tRNA	RF00005	scaffold9884	34548	34475	-	4.90E-14	tRNA
tRNA	RF00005	scaffold9884	34459	34387	-	8.70E-13	tRNA
tRNA	RF00005	scaffold9884	35066	34993	-	1.10E-11	tRNA
tRNA	RF00005	scaffold9884	34973	34884	-	7.80E-09	tRNA
tRNA	RF00005	scaffold9884	34704	34579	-	7.00E-07	tRNA
tRNA	RF00005	scaffold11157	66	139	+	1.40E-15	tRNA
tRNA	RF00005	scaffold11157	2906	2978	+	4.00E-14	tRNA
tRNA	RF00005	scaffold11157	2378	2467	+	1.40E-10	tRNA
tRNA	RF00005	scaffold11157	2798	2882	+	2.20E-10	tRNA
tRNA	RF00005	C2393834	7	78	+	6.70E-16	tRNA
tRNA	RF00005	C2394592	32	101	+	3.60E-12	tRNA
tRNA	RF00005	C2395210	47	101	+	5.40E-05	tRNA
tRNA	RF00005	C2395426	80	7	-	6.40E-13	tRNA
tRNA	RF00005	C2396590	42	101	+	0.0089	tRNA
tRNA	RF00005	C2601192	85	13	-	7.70E-17	tRNA
tRNA	RF00005	C2601192	401	328	-	2.40E-16	tRNA
tRNA	RF00005	C2601192	220	148	-	2.10E-14	tRNA
tRNA	RF00005	C2601192	304	232	-	8.50E-14	tRNA
tRNA	RF00005	C2726737	179	249	+	6.00E-13	tRNA
tRNA	RF00005	C2726737	28	98	+	8.30E-13	tRNA
tRNA	RF00005	C2726737	289	361	+	1.30E-10	tRNA
tRNA-Sec	RF01852	scaffold9884	34973	34885	-	1.10E-06	tRNA Selenocysteine transfer RNA
tRNA-Sec	RF01852	scaffold6077	83631	83711	+	3.40E-06	tRNA Selenocysteine transfer RNA
tRNA-Sec	RF01852	scaffold6925	65407	65338	-	3.90E-05	tRNA Selenocysteine transfer RNA
tRNA-Sec	RF01852	scaffold7868	2604	2521	-	1.20E-05	tRNA Selenocysteine transfer RNA
tRNA-Sec	RF01852	scaffold7868	2907	2825	-	6.90E-05	tRNA Selenocysteine transfer RNA
tRNA-Sec	RF01852	scaffold9445	3418	3336	-	0.0015	tRNA Selenocysteine transfer RNA

tRNA-Sec	RF01852	scaffold11157	2798	2881	+	1.80E-05	tRNA Selenocysteine transfer RNA
tRNA-Sec	RF01852	scaffold11157	2907	2976	+	7.50E-05	tRNA Selenocysteine transfer RNA
tRNA-Sec	RF01852	C2393834	6	78	+	5.40E-07	tRNA Selenocysteine transfer RNA
tRNA-Sec	RF01852	C2398740	78	3	-	0.0005	tRNA Selenocysteine transfer RNA
tRNA-Sec	RF01852	C2726737	288	361	+	0.0075	tRNA Selenocysteine transfer RNA
Trp_leader	RF00513	C2389182	9	98	+	0.0099	Tryptophan operon leader
Trp_leader	RF00513	C2412058	108	54	-	0.0056	Tryptophan operon leader
uc_338	RF02271	scaffold6883	1395	1590	+	0.0041	TUC338
U1	RF00003	scaffold1219	50660	50770	+	0.00047	U1 spliceosomal RNA
U1	RF00003	scaffold5596	28143	28315	+	0.00039	U1 spliceosomal RNA
U11	RF00548	scaffold5676	20763	20633	-	3.90E-19	U11 spliceosomal RNA
U12	RF00007	scaffold557	20367	20520	+	7.10E-25	U12 minor spliceosomal RNA
U12	RF00007	scaffold7605	1122	970	-	0.00074	U12 minor spliceosomal RNA
U2	RF00004	scaffold819	84665	84718	+	4.70E-08	U2 spliceosomal RNA
U2	RF00004	scaffold2136	4546	4374	-	2.90E-20	U2 spliceosomal RNA
U2	RF00004	scaffold7149	4469	4517	+	5.00E-05	U2 spliceosomal RNA
U2	RF00004	scaffold8363	24240	24194	-	7.30E-06	U2 spliceosomal RNA
U2	RF00004	C2545250	234	264	+	4.20E-06	U2 spliceosomal RNA
U4	RF00015	scaffold5599	9030	8895	-	6.30E-10	U4 spliceosomal RNA
U4atac	RF00618	scaffold5555	70644	70515	-	1.40E-16	U4atac minor spliceosomal RNA
U5	RF00020	scaffold382	15250	15365	+	5.10E-07	U5 spliceosomal RNA
U6	RF00026	scaffold1407	9357	9419	+	0.0076	U6 spliceosomal RNA
U6	RF00026	scaffold2215	3962	4027	+	0.0012	U6 spliceosomal RNA
U6	RF00026	scaffold3479	548	486	-	5.10E-07	U6 spliceosomal RNA
U6	RF00026	scaffold3863	2775	2852	+	9.40E-06	U6 spliceosomal RNA
U6	RF00026	scaffold4687	7341	7239	-	0.00057	U6 spliceosomal RNA
U6	RF00026	scaffold5050	7552	7617	+	4.70E-05	U6 spliceosomal RNA
U6	RF00026	scaffold9624	80	5	-	7.60E-09	U6 spliceosomal RNA
U6	RF00026	scaffold10188	599	623	+	0.001	U6 spliceosomal RNA
U6	RF00026	C2449648	69	130	+	2.70E-11	U6 spliceosomal RNA
U6	RF00026	C2461163	38	130	+	4.30E-08	U6 spliceosomal RNA
U6	RF00026	C2570874	280	343	+	0.00032	U6 spliceosomal RNA
U6	RF00026	C2639021	663	764	+	0.00076	U6 spliceosomal RNA
U6	RF00026	C2639021	823	777	-	0.0023	U6 spliceosomal RNA
U6atac	RF00619	scaffold2337	92501	92412	-	3.40E-21	U6atac minor spliceosomal RNA
U6atac	RF00619	scaffold5941	8030	8142	+	0.0063	U6atac minor spliceosomal RNA

UPSK	RF00390	scaffold10628	3587	3565	-	0.0071	UPSK RNA
UPSK	RF00390	C2443546	44	63	+	0.0087	UPSK RNA
WT1-AS_7	RF02209	scaffold3829	1836	1696	-	0.0079	WT1 antisense RNA conserved region 7
XIST_intron	RF02266	C2443664	67	117	+	0.0068	XIST 3' intron conserved motif
Yar_3	RF02087	C2527221	2	53	+	0.004	Yellow-achaete intergenic RNA conserved regic
ykoK	RF00380	scaffold6925	49910	50085	+	1.60E-29	ykoK leader

Table S3. A total of 54 predicted proteins had significant matches using the COesterase HMM model from the PFAM database.

H. hampei protein	PFAM name	PFAM ID	e-value
evmmodelscaffold31553	COesterase	PF00135.23	7.20E-167
evmmodelscaffold9868	COesterase	PF00135.23	1.40E-144
evmmodelscaffold12281	COesterase	PF00135.23	7.10E-143
evmmodelscaffold22221	COesterase	PF00135.23	4.70E-138
evmmodelscaffold12283	COesterase	PF00135.23	1.60E-136
evmmodelscaffold86116	COesterase	PF00135.23	5.90E-135
evmmodelscaffold8692	COesterase	PF00135.23	1.10E-134
evmmodelscaffold1011	COesterase	PF00135.23	2.60E-133
evmmodelscaffold12282	COesterase	PF00135.23	1.00E-132
evmmodelscaffold4214	COesterase	PF00135.23	2.60E-131
evmmodelscaffold50432	COesterase	PF00135.23	1.40E-129
evmmodelscaffold20746	COesterase	PF00135.23	2.60E-128
evmmodelscaffold51801	COesterase	PF00135.23	9.50E-128
evmmodelscaffold26061	COesterase	PF00135.23	2.00E-127
evmmodelscaffold21622	COesterase	PF00135.23	7.20E-127
evmmodelscaffold7084	COesterase	PF00135.23	1.60E-126
evmmodelscaffold67111	COesterase	PF00135.23	5.60E-126
evmmodelscaffold87511	COesterase	PF00135.23	1.20E-125
evmmodelscaffold38928	COesterase	PF00135.23	8.80E-125
evmmodelscaffold46255	COesterase	PF00135.23	2.10E-124
evmmodelscaffold55223	COesterase	PF00135.23	2.10E-122
evmmodelscaffold47451	COesterase	PF00135.23	2.10E-122

evmmodelscaffold64441	COesterase	PF00135.23	1.50E-121
evmmodelscaffold6682	COesterase	PF00135.23	4.20E-121
evmmodelscaffold38484	COesterase	PF00135.23	8.10E-121
evmmodelscaffold1171	COesterase	PF00135.23	1.30E-120
evmmodelscaffold28619	COesterase	PF00135.23	4.80E-120
evmmodelscaffold28618	COesterase	PF00135.23	1.30E-119
evmmodelscaffold2382	COesterase	PF00135.23	2.40E-115
evmmodelscaffold50961	COesterase	PF00135.23	1.60E-112
evmmodelscaffold98932	COesterase	PF00135.23	1.30E-108
evmmodelscaffold26282	COesterase	PF00135.23	4.40E-108
evmmodelscaffold51803	COesterase	PF00135.23	2.10E-105
evmmodelscaffold21801	COesterase	PF00135.23	4.50E-103
evmmodelscaffold221	COesterase	PF00135.23	1.20E-95
evmmodelscaffold27053	COesterase	PF00135.23	7.10E-84
evmmodelscaffold2381	COesterase	PF00135.23	1.60E-82
evmmodelscaffold13242	COesterase	PF00135.23	4.00E-81
evmmodelscaffold100952	COesterase	PF00135.23	2.20E-72
evmmodelscaffold7983	COesterase	PF00135.23	3.40E-67
evmmodelscaffold14072	COesterase	PF00135.23	1.20E-65
evmmodelscaffold7982	COesterase	PF00135.23	2.10E-48
evmmodelscaffold49871	COesterase	PF00135.23	2.00E-28
evmmodelscaffold26281	COesterase	PF00135.23	2.10E-28
evmmodelscaffold31723	COesterase	PF00135.23	2.70E-27
evmmodelscaffold31711	COesterase	PF00135.23	1.30E-26
evmmodelscaffold34911	COesterase	PF00135.23	6.50E-18
evmmodelscaffold54841	COesterase	PF00135.23	1.40E-13
evmmodelscaffold98931	COesterase	PF00135.23	3.10E-09

evmmodelC25773661	COesterase	PF00135.23	0.00049
evmmodelscaffold425513	COesterase	PF00135.23	0.005
evmmodelscaffold47122	COesterase	PF00135.23	0.0054
evmmodelscaffold3394	COesterase	PF00135.23	0.037
evmmodelscaffold33334	COesterase	PF00135.23	0.11
